# Moderate static magnetic fields enhance antitumor CD8^+^ T cell function by promoting mitochondrial respiration

**DOI:** 10.1038/s41598-020-71566-x

**Published:** 2020-09-03

**Authors:** Xiaoyan Zhu, Yan Liu, Xianxia Cao, Haifeng Liu, Ao Sun, Hao Shen, Jingyao Zhao, Ronghong Li, Ligang Wu, Zhicai Fang, Hui Wang, Qiwei Zhai

**Affiliations:** 1 State Key Laboratory of Cell Biology, Shanghai Institute of Biochemistry and Cell Biology, Center for Excellence in Molecular Cell Science, Chinese Academy of Science, University of Chinese Academy of Sciences, Shanghai, 200031, China; 2 School of Life Science and Technology, Shanghai Tech University, Shanghai, 201210 China; 3 Heye Health Technology Co., Ltd., Zhejiang, 313300 China; 4 Key Laboratory of Nutrition and Metabolism, CAS Center for Excellence in Molecular Cell Sciences, Institute for Nutritional Sciences, Shanghai Institutes for Biological Sciences, Chinese Academy of Sciences, Shanghai, 200031 China

**Keywords:** Adaptive immunity, Tumour immunology, Immunology

## Abstract

With the discovery of magnetoreceptor mechanisms in animals, it materialized the novel applications of controlling cell and animal behaviors using magnetic fields. T cells have shown to be sensitive to magnetic fields. Here, we reported that exposure to moderate SMFs (static magnetic fields) led to increased granule and cytokine secretion as well as ATP production and mitochondrial respiration from CD8^+^ T cells. These effects were inhibited by knocking down the *Uqcrb* and *Ndufs6* genes of mitochondrial respiratory chain, whose transcriptions were regulated by candidate magnetoreceptor genes *Isca1* and *Cry1*/*Cry2*. SMF exposure also promoted CD8^+^ T cell granule and cytokine secretion and repressed tumor growth in vivo. SMFs enhanced CD8^+^ T cell cytotoxicity, and the adoptive transfer into tumor-bearing mice resulted in enhanced antitumor effects. Collectively, our study suggests that moderate SMFs enhance CD8^+^ T cell cytotoxicity by promoting mitochondrial respiration and promoted the antitumor function of CD8^+^ T cells.

## Introduction

T cells are critical components of the adaptive immune system. T cells can be divided into CD4^+^ T cells and CD8^+^ T cells based on the surface expression of CD4 and CD8; these cells are linked to different T cell functions^1^. CD4^+^ T cells are also called helper T cells, which support the activity of other immune cells by releasing T cell cytokines such as IFNγ, IL-4, IL-17 and TNFα^2^. CD8^+^ T cells are also called cytotoxic T lymphocytes (CTLs), which mediate the direct killing of infected, damaged, and dysfunctional cells and play critical roles in antitumor immunity^3^. The cytolytic targeted killing of cancer cells by CTLs requires perforin-mediated release of granzymes, mainly granzyme B, from cytotoxic granules^4^. In addition, CTLs can eliminate cancer cells or virally infected cells by secreting cytokines such as IFNγ and TNFα^3,5^.

The magnetic field is an important physical factor similar to the temperature and pressure existing in the environment. Depending on intensity, static magnetic fields (SMFs) can be classified as weak (< 1 mT), moderate (1 mT–1 T), strong (1–5 T), and ultrastrong (> 5 T)^6^. Many animals sense geomagnetic field (25 − 65 μT) for the purpose of orientation or to navigate and migrate over long distances^7^. Cryptochromes (Cry) have been reported to be the best biochemical magnetoreceptor candidates in animals such as birds and *Drosophila*, which mediate light-dependent magnetosensitivity via a radical-pair reaction^8–10^. Another magnetoreceptor candidate, an iron-sulfur cluster assembly protein 1 (Isca1), has been reported to form a rod-like complex with Cry and works like a biocompass^11^.

In addition, a lot of recent studies have shown that SMFs have various cellular effects on cells^12–16^. It seems that strong and ultrastrong SMFs have harmful effects on normal cells, such as altering cleavage planes, changing the orientation and morphology of mitotic spindles and causing DNA damage^15,17,18^. However, moderate SMFs showed much fewer harmful effects on normal cells and exhibited great potential in changing cell behaviors, including altering stem cell proliferation, promoting osteoclast differentiation, impairing cancer cell proliferation, enhancing oligodendrocyte differentiation and promoting neurotrophic factor secretion^12–14,19^. With the discovery of magnetoreceptor genes and related mechanism models^9,11,20,21^, it materialized the novel applications of controlling cell and animal behaviors based on magnetoreceptor mechanisms.

T cells have presented to be sensitive to SMFs. Exposure to strong SMFs (> 3 T) has been reported to induce genotoxic effects and increase apoptosis of human T lymphocytes^18,22^. Moreover, exposure to 1.5 T SMFs leads to suppress the release of cytokines IL-6, IL-8, and TNF-α, but assists the production of IL-10 of human lymphocytes and macrophages^23^. These results suggest that strong SMFs can change the behavior of T cells. However, the underlined mechanism of SMFs influencing T cell behavior is largely unknown, and lacks in vivo evidence.

In the present study, we studied the cellular effects of moderate SMFs on T cells using magnetic intensity of 0.3 T and 0.6 T in mouse model. We found that exposure to moderate SMFs led to increased granule and cytokine secretion from CD8^+^ T cells as well as elevated ATP production and mitochondrial respiration. These effects were inhibited by knocking down the *Uqcrb* or *Ndufs6* genes, which are related to mitochondrial respiratory electron transport chain. In addition, knocking down the magnetoreceptor genes *Isca1* or *Cry1*/*Cry2* led to reduced transcription of *Uqcrb* and *Ndufs6*, and also reversed the increased granule and cytokine secretion and ATP production of SMF treated CD8^+^ T cells. Moreover, SMFs enhanced CD8^+^ T cell cytotoxicity, and adoptive transfer of SMF-treated CTLs into tumor-bearing mice resulted in enhanced antitumor effects. Taken together, our study not only elucidate the molecular mechanism of SMFs in enhancing CD8^+^ T cell cytotoxicity by promoting mitochondrial metabolism, but also provides a new approach of promoting the antitumor function of CD8^+^ T cells using a physical method.

## Results

### Moderate SMF exposure enhances CD8^+^ T cell granule and cytokine secretion

To determine whether moderate SMFs affect T cell function, we first examined T cell activation upon T cell receptor (TCR) stimulation at different time points. Purified CD4^+^ T cells or CD8^+^ T cells from the spleens of C57/B6 mice were cultured in plates that were placed on permanent magnets with a magnetic intensity of 0.3 T or 0.6 T (as shown in Supplementary Fig. S1); control cells were cultured without magnets. The surface expression levels of T cell activation markers, including CD69, CD44 and CD25, were analyzed at 24 h, 48 h and 72 h after TCR stimulation. In addition, unstimulated cells were also compared. The results indicated that moderate SMFs did not affect the surface expression levels of T cell activation marker of both CD4^+^ T cells (Supplementary Fig. S2A-D) and CD8^+^ T cells (Supplementary Fig. S3A-D) with no stimulation. In the case of stimulation, the expression level of CD44 of CD4^+^ T cells revealed by the statistical analysis of MFI (mean fluorescence intensity) was higher in 0.3 T SMF-treated cells than in control cells at all time points (Supplementary Fig. S2E–H). However, for CD8^+^ T cells, CD25 was higher in 0.3 T SMF-treated samples at 48 h and 72 h (Supplementary Fig. S3E,G and H).

**Figure. 1:**
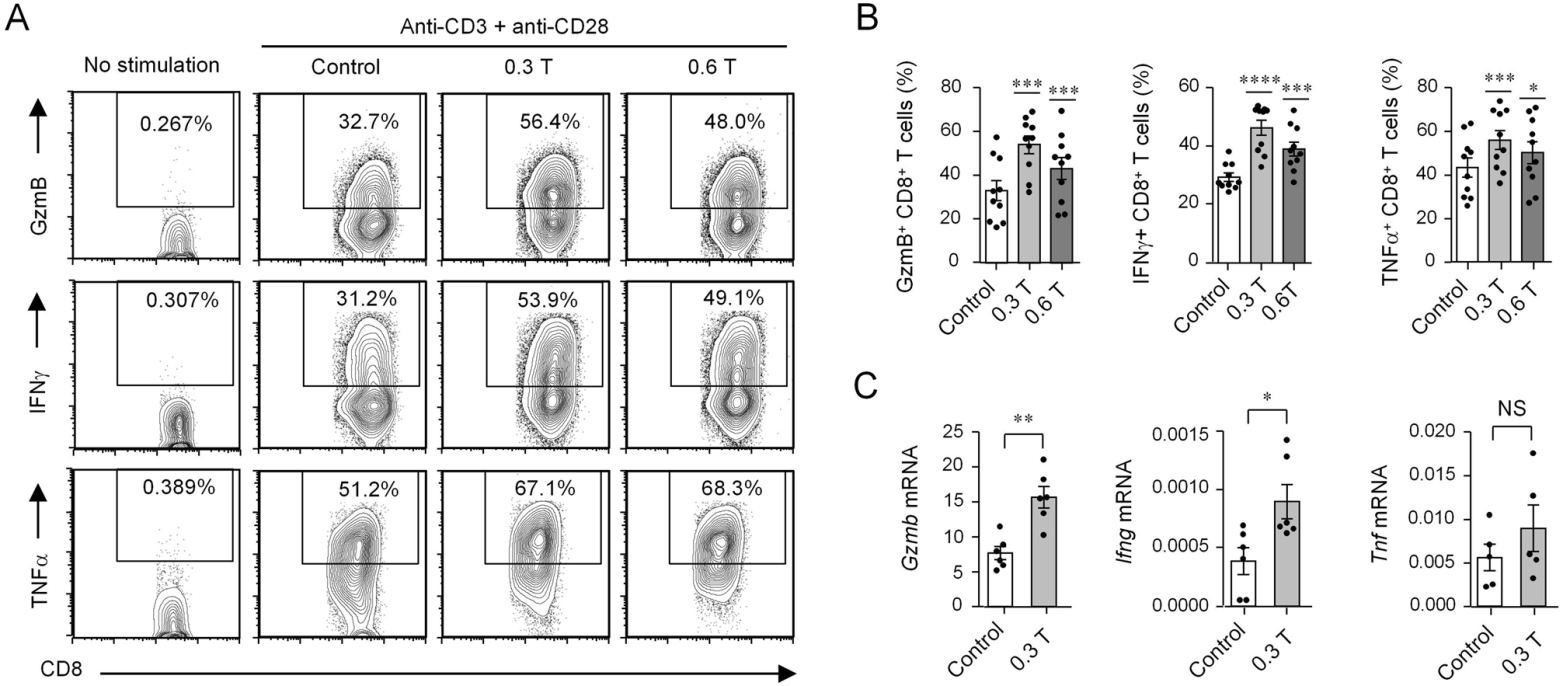
Moderate SMFs enhance CD8^+^ T cell granule and cytokine secretion at 72 h stimulation. (**A**) Cytokine/granule production of stimulated mouse CD8^+^ T cells analyzed by flow cytometry. Cell samples were stimulated with anti-CD3 and anti-CD28 antibodies in the presence of 0.3 T or 0.6 T permanent magnets, and control cells were treated without magnets. Cell samples with no stimulation were used to show the baseline of cytokine secretion. (**B**) Percentage statistics for the expression of GzmB, IFNγ and TNFα of CD8^+^ T cells stimulated for 72 h (**B**, n = 10). (**C**) Relative transcriptional levels of *Gmzb*, *Tnfa*, and *Ifng* in 0.3 T SMF-treated and control CD8^+^ T cells (n = 6). The cell samples were stimulated with anti-CD3 and anti-CD28 antibodies for 72 h. All the relative transcription levels of target genes were normalized to β-actin. Data were analyzed by Student’s t-test; NS, no significance, *p < 0.05; **p < 0.01; ***p < 0.001, ****p < 0.0001. Error bars indicate the s.e.m. Data are representative of or combined from at least three independent experiments.

Next, we analyzed the effects of SMFs on cytokine secretion from both CD4^+^ T cells and CD8^+^ T cells. The cytokine production of purified CD4^+^ T cells showed no significant changes between SMF-treated cells and control cells at all time points (Supplementary Fig. S4). In contrast, the granule GzmB and cytokine production including IFNγ and TNFα from isolated CD8^+^ T cells cultured from 72 h after stimulation was significantly increased in both the 0.3 T and 0.6 T SMF-treated cells compared with the control cells (Fig. 1A,B), although there were no predominant changes at 24 h and 48 h after stimulation (Supplementary Fig. S5). Moreover, the 0.3 T SMF showed a more significant change in CD8^+^ T cell cytokine secretion than the 0.6 T SMF (Fig. 1A,B). To exclude if the effects were caused by foreign substance with no magnetism of permanent magnets, we also compared the effects of the sham group, with cell samples placed on metal blocks with no magnetism of the same size of 0.3 T permanent magnets. The results showed that there was no significant changes in CD8^+^ T cell cytokine secretion between the control group (without magnets) and the sham group (Supplementary Fig. S6A, B). In addition, the granule GzmB and cytokine production of CD8^+^ T cells was significantly increased in 0.3 T magnet-treated cells compared with the sham group as well as the control group (Supplementary Fig. S6A, B). Consequently, the expression levels of gene products commonly found in CD8^+^ T cell granules and cytokine secretion were also validated. The mRNA expression levels of *Gmzb* and *Ifnr* were upregulated in 0.3 T SMF-treated cells compared with those in control cells; however, *Tnf* gene expression showed no significant change (Fig. 1C).

We further examined whether 0.3 T SMF affected the proliferation and survival of CD8^+^ T cells. We assessed the cell proliferation of CD8^+^ T cells by the CFSE labeling assay; SMF-treated cells showed a comparable ratio of cycling cells as that of control cells (Supplementary Fig. S7A). Moreover, no significant changes in T cell apoptosis and death were detected between SMF-treated cells and control cells (Supplementary Fig. S7B, C). Taken together, the above data indicated that SMFs promoted CD8^+^ T cell granule and cytokine secretion without affecting cell proliferation or survival, and SMFs had no obvious effects on the cytokine production of CD4^+^ T cells.

### Moderate SMF exposure promotes the transcription of genes related to mitochondrial respiratory electron transport chain

**Figure. 2:**
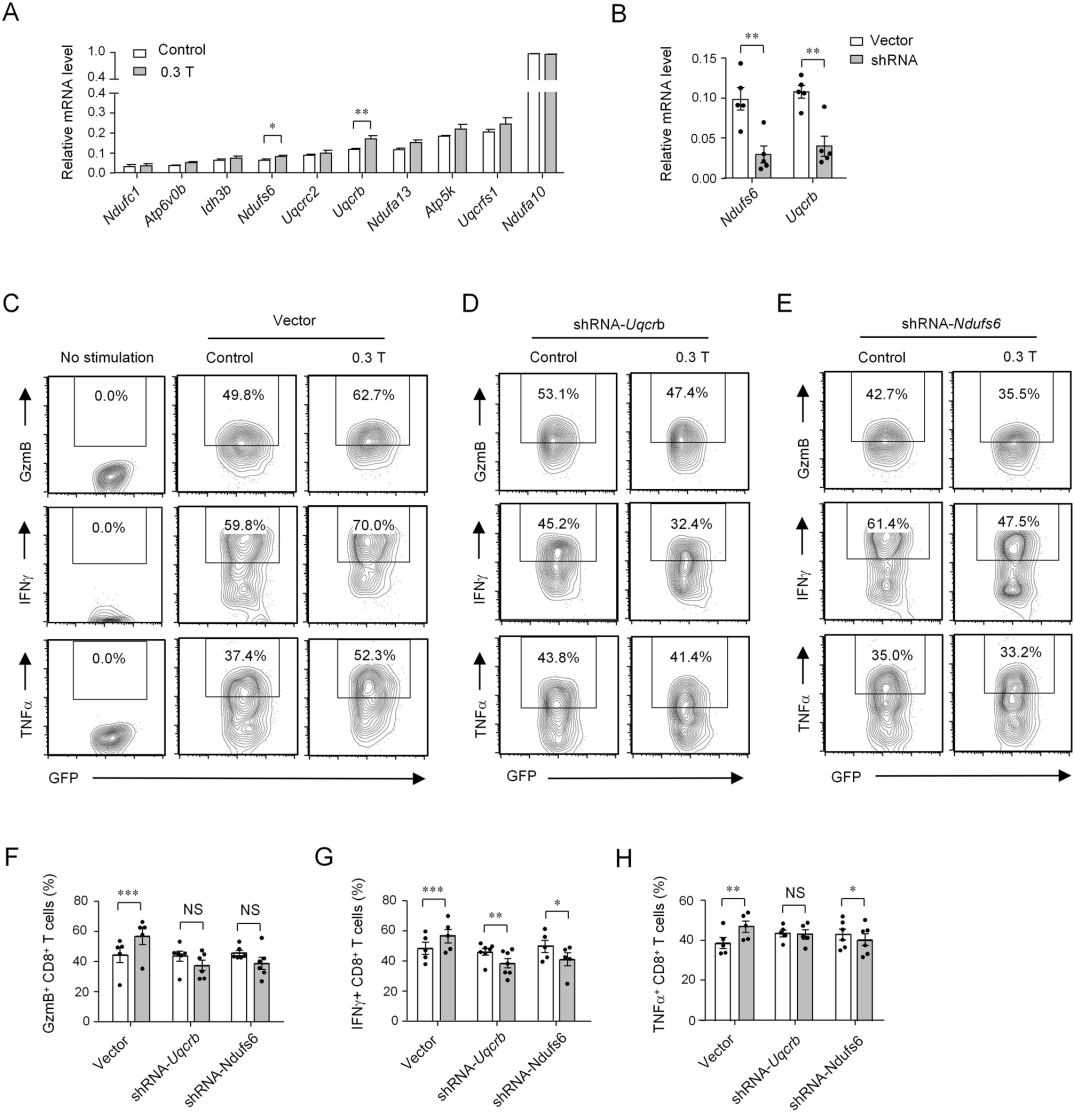
Moderate SMFs enhance the granule and cytokine secretion of CD8^+^ T cells by modulating the expression of genes related to mitochondrial respiratory electron transport chain. (**A**) Relative transcriptional levels of genes related to mitochondrial respiratory electron transport chain in 0.3 T SMF-treated CD8^+^ T cells stimulated with anti-CD3 and anti-CD28 antibodies for 72 h and control cells without magnets (n = 3–7). (**B**) Analysis of *Uqcrb* and *Ndufs6* mRNA levels in control and knockdown CD8^+^ T cells (n = 5). All the relative transcription levels of target genes were normalized to β-actin. (**C**–**E)** Cytokine/granule production of knockdown CD8^+^ T cells cultured in the presence or absence of 0.3 T magnets analyzed by flow cytometry. Cell samples were stimulated with anti-CD3 and anti-CD28 antibodies in the presence of 0.3 T magnets, and control cells were treated without magnets. Cell samples with no stimulation were used to show the baseline of cytokine secretion. (**F**, **G** and **H**) Percentage statistics for the expression of GzmB (**F**), IFNγ (**G**) and TNFα (**H**) of knockdown CD8^+^ T cells (n = 5–7). Cells transfected with shRNA-*Uqcrb* or shRNA-*Ndufs6* were compared with Vector. Data were analyzed by Student’s t-test; NS, no significance, *p < 0.05; **p < 0.01, ***p < 0.001. Error bars indicate the s.e.m. Data are representative of or combined from at least three independent experiments.

Next, we sought to determine the underlying mechanism of the enhanced effector function of mouse CD8^+^ T cells induced by SMFs. Cell samples, including unstimulated fresh CD8^+^ T cells and stimulated CD8^+^ T cells with or without exposure to 0.3 T magnets, were subjected to RNA-Seq-based transcriptome analysis (data available on GEO: GSE113858). Two main clusters of genes with a fold change ≥ 2 were identified by functional enrichment analysis. Cluster 1 contained 198 genes that mainly participated in mRNA metabolic processes and RNA processing (Supplementary Fig. S8A). Cluster 2 contained 24 genes that participated in adenosine triphosphate (ATP) synthesis and respiratory electron transport chain in mitochondria (Supplementary Fig. S8A). The genes of cluster 2 attracted our attention because genes in the respiratory electron transport chain include various genes encoding iron-sulfur cluster proteins, which possess strong magnetic properties and are considered candidates for magnetoreceptor proteins^11,20^. Specifically, 10 genes from cluster 2 which were closely related to mitochondrial respiratory electron transport chain were obviously upregulated in SMF-treated cells compared with those in control cells based on two replicates of RNA-seq analyses (Supplementary Fig. S8B). Therefore, we speculated that SMFs enhanced CD8^+^ T cell granule and cytokine secretion by upregulating the expression of gene related to mitochondrial respiratory electron transport chain. Consequently, the gene expression levels of these 10 genes were validated by real-time PCR analysis. The results revealed that, among these genes, *Uqcrb* and *Ndufs6* were significantly upregulated in SMF-treated cells compared with control cells (Fig. 2A).

To verify whether *Uqcrb* or *Ndufs6* gene upregulation is required for 0.3 T SMF-induced enhanced granule and cytokine secretion in CD8^+^ T cells, we used a shRNA expression vector system to perform a knockdown assay. The knockdown efficiency of *Uqcrb* and *Ndufs6* in primary CD8^+^ T cells was tested by real-time PCR (Fig. 2B). Once *Uqcrb* or *Ndufs6* was efficiently knocked down, the enhanced CD8^+^ T cell granule and cytokine secretion in 0.3 T SMF-treated cells were effectively inhibited (Fig. 2C – H). Both of *Uqcrb* and *Ndufs6* gene knockdown led to decreased secretion of IFNγ in SMF-treated cells, and *Ndufs6* gene knockdown also led to decreased secretion of TNFα (Fig. 2C − H). These data suggested that 0.3 T SMF enhanced CD8^+^ T cell granule and cytokine secretion presumably by upregulating the expression of *Uqcrb* and *Ndufs6*.

### Moderate SMF exposure improves ATP production and mitochondrial respiration

**Figure. 3:**
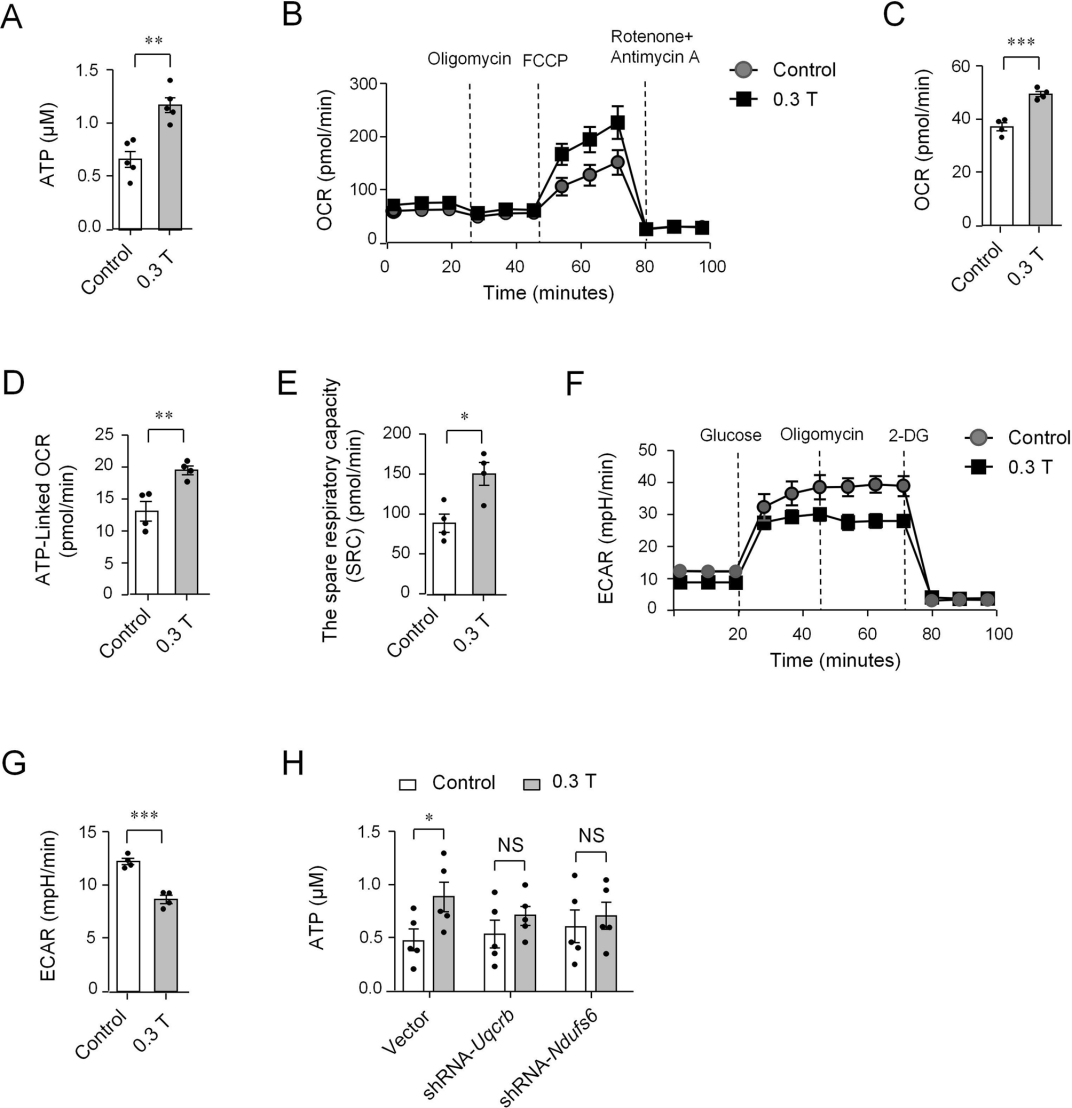
Moderate SMFs improves ATP production and mitochondrial respiration of CD8^+^ T cells. (**A**) The relative intracellular ATP concentration was measured in CD8^+^ T cells stimulated with anti-CD3 and anti-CD28 antibodies for 72 h (n = 5). (**B**) OCR of stimulated CD8^+^ T cells at baseline and in response to oligomycin, FCCP, and rotenone with antimycin as detected by the Seahorse MitoStress assay. (**C**) Baseline OCR of stimulated CD8^+^ T cells (n = 4). (**D**) ATP-linked OCR (baseline OCR minus the OCR in the presence of oligomycin) of stimulated CD8^+^ T cells (n = 4). (**E**) The spare respiratory capacity (SRC) of stimulated CD8^+^ T cells (n = 4). (**F**) ECAR of stimulated CD8^+^ T cells at baseline and in response to glucose, oligomycin, and 2-DG as detected by the Seahorse MitoStress assay. (**G**) Baseline ECAR of stimulated CD8^+^ T cells (n = 4). (**H**) ATP concentration of knockdown CD8^+^ T cells compared with that in cells transfected with vectors in the presence or absence of magnets (n = 5). Cell samples were treated with 0.3 T magnets, and samples treated without magnets were used as controls. Data were analyzed by Student’s t-test; NS, no significance, *p < 0.05; **p < 0.01; ***p < 0.001. Error bars indicate the s.e.m. Data are representative of or combined from at least three independent experiments.

The aforementioned data demonstrated that 0.3 T SMF promoted the expression of genes involved in ATP synthesis and respiratory electron transport chain in CD8^+^ T cells. This suggested that 0.3 T SMF may influence ATP production and mitochondrial respiration in CD8^+^ T cells. Thus, we assessed the concentration of ATP in CD8^+^ T cells in the presence and absence of 0.3 T SMF. The level of intracellular ATP was increased in SMF-treated CD8^+^ T cells compared with that in control cells cultured without SMFs (Fig. 3A). To further verify whether 0.3 T SMF influences mitochondrial respiration in CD8^+^ T cells, we next examined the oxygen consumption rate (OCR) in CD8^+^ T cells. The OCR was also elevated in SMF-treated CD8^+ ^T cells compared with that in control cells (Fig. 3B,C). Increased ATP levels and an ATP production rate, which is determined by subtracting the OCR in the presence of oligomycin (an ATP synthase inhibitor) from the OCR at baseline, were also observed in SMF-treated CD8^+^ T cells compared with those in control cells (Fig. 3B,D). In addition, the spare respiratory capacity (SRC) was also elevated (Fig. 3B,E). In contrast, analysis of the extracellular acidification rate (ECAR) in response to glucose showed that ECAR was decreased in SMF-treated CD8^+^ T cells compared with that in control cells (Fig. 3F,G). Moreover, the aforementioned data revealed that 0.3 T SMF enhanced CD8^+ ^T cell granule and cytokine secretion mainly by regulating the *Uqcrb* and *Ndufs6* genes of the respiratory electron transport chain. We wondered whether these two genes also regulated the ATP levels in CD8^+ ^T cells. The results of the knockdown assay revealed that knocking down either *Uqcrb* or *Ndufs6* can inhibit the increased ATP levels in SMF-treated CD8^+ ^T cells (Fig. 3H).

### Moderate SMF promotes the transcription of Uqcrb and Ndufs6 via *candidate magnetoreceptor genes*

**Figure. 4:**
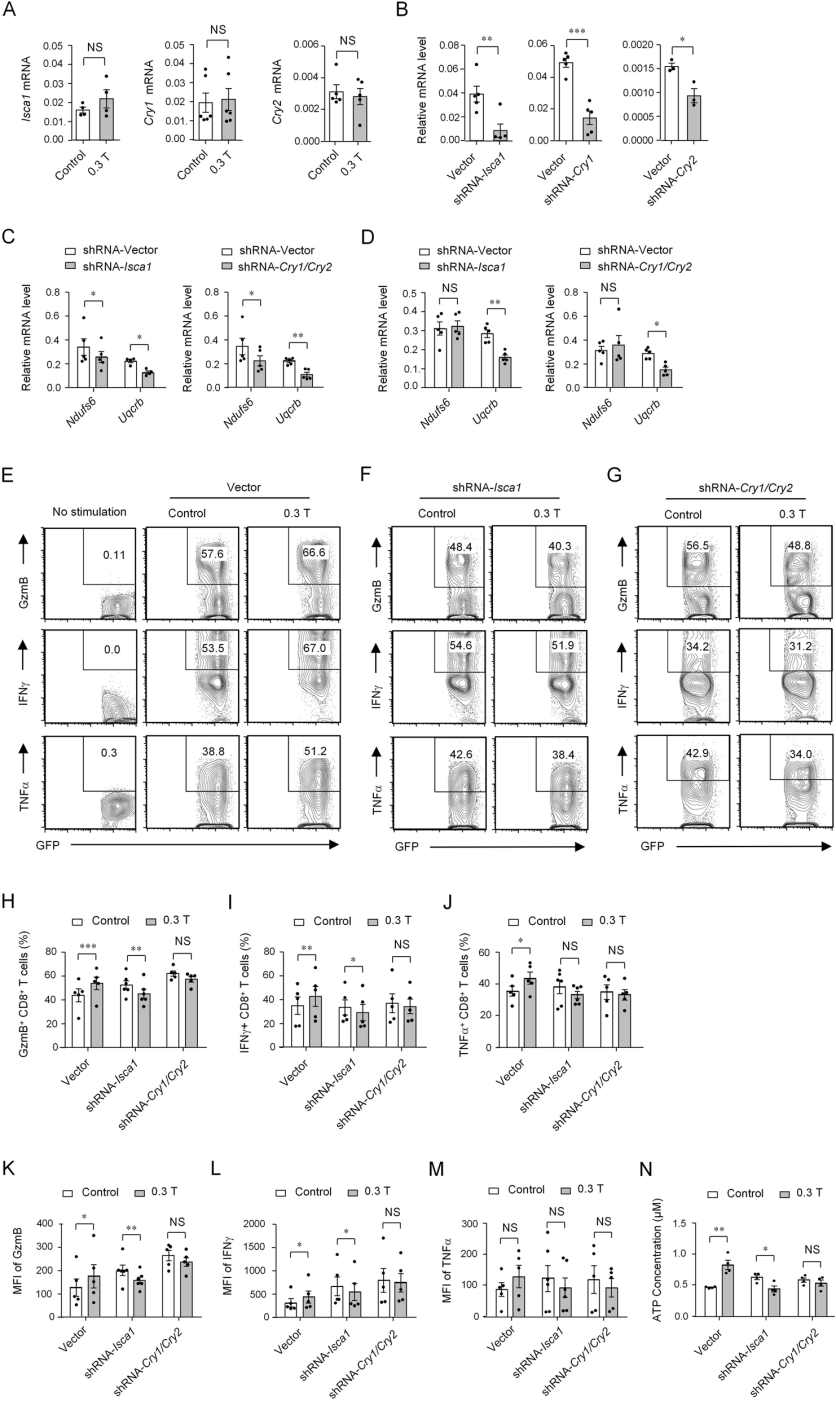
Candidate magnetoreceptor genes involve in modulating the transcription of *Uqcrb* and *Ndufs6*. (**A**) Relative transcriptional levels of candidate magnetoreceptor genes *Isca1*, *Cry1* and *Cry2* in 0.3 T SMF-treated stimulated CD8^+^ T cells and control cells (n = 4–6). (**B**) Analysis of mRNA levels of *Isca1*, *Cry1* and *Cry2* in control and knockdown CD8^+^ T cells (n = 4–5). (**C** and **D**) Analysis of *Uqcrb* and *Ndufs6* mRNA levels in control and *Isca1* or *Cry1*/*Cry2* knockdown CD8^+^ T cells treated without magnets (**C**) or with 0.3 T magnets (**D**) (n = 5). All the relative transcription levels of target genes were normalized to β-actin. (**E**–**G**) Cytokine/granule production of knockdown CD8^+^ T cells cultured in the presence or absence of 0.3 T magnets analyzed by flow cytometry. Cell samples were stimulated with anti-CD3 and anti-CD28 antibodies in the presence of 0.3 T magnets, and control cells were treated without magnets. Cell samples with no stimulation were used to show the baseline of cytokine secretion. (**H–M**) Percentage (H–J) and MFI (K–M) statistics for the expression of GzmB, IFNγ and TNFα of knockdown CD8^+^ T cells (n = 5–6). Cells transfected with shRNA-*Isca1* or shRNA-*Cry1/Cry2* were compared with Vector. (**N**) ATP concentration of knockdown CD8^+^ T cells compared with that in cells transfected with vectors in the presence or absence of magnets (n = 4). Data were analyzed by Student’s t-test; NS, no significance, *p < 0.05; **p < 0.01; ***p < 0.001. Error bars indicate the s.e.m. Data are representative of or combined from at least three independent experiments.

Because *Isca1* and *Cry1/Cry2* were identified as candidate magnetoreceptor genes^9,11,20,24^, we are curious if SMFs promotes the transcription of *Uqcrb* or *Ndufs6* via these candidate magnetoreceptor genes. Our RNA-seq results indicated that *Isca1* and *Cry1/Cry2* were not included in the significant changing genes in 0.3 T SMF-treated CD8^+^ T cells compared with control cells (Supplementary Fig. S9A,B). Then we confirmed the expression levels of these candidate magnetoreceptor genes in the presence or absence of magnets. The expression of these genes exhibited no significant changes between 0.3 T SMF-treated cells and control cells (Fig. 4A), which was consistent with our RNA-seq data. Next, we performed knockdown assays of these candidate magnetoreceptor genes to detect if it will prevent the promoted transcription level of *Uqcrb* or *Ndufs6* in SMF treated CD8^+^ T cells. The knockdown efficiency was detected as shown in Fig. 4B. The transcription levels of both *Uqcrb* and *Ndufs6* were predominated reduced by knocking down either *Isca1* or *Cry1/Cry2* in the absence of 0.3 T magnets (Fig. 4C). In the presence of magnets, however, knocking down *Isca1* or *Cry1/Cry2* can only reverse the promoted transcription of *Uqcrb* (Fig. 4D), but has weak impacts on *Ndufs6*. This may be caused by the upregulation of *Uqcrb* and *Ndufs6* in the presence of magnets (Fig. 2A).

Then we examined the impacts of knocking down candidate magnetoreceptor genes on granule and cytokine secretion as well as ATP production of CD8^+^ T cells. The results revealed that either *Isca1* or *Cry1/Cry2* gene knockdown can prevent the enhanced CD8^+^ T cell granule and cytokine secretion in 0.3 T SMF-treated cells (Fig. 4E–M). Specially, *Isca1* gene knockdown reversed the enhanced GzmB and IFNγ secretion of 0.3 T SMF-treated cells (Fig. 4H−M). Moreover, the results of the knockdown assay showed that knocking down *Isca1* or *Cry1/Cry2* can inhibit the increased ATP levels in 0.3 T SMF-treated CD8^+^ T cells, and *Isca1* had more obvious effects (Fig. 4N). Together, these data suggested that 0.3 T SMF promotes the transcription of *Uqcrb* or *Ndufs6* via candidate magnetoreceptor genes including *Isca1* and *Cry1/Cry2*, whose knocking down can prevent the increased granule and cytokine secretion as well as ATP production of 0.3 T SMF-treated CD8^+ ^T cells.

### Moderate SMF exposure enhances CD8^+^ T cell cytotoxicity

**Figure 5:**
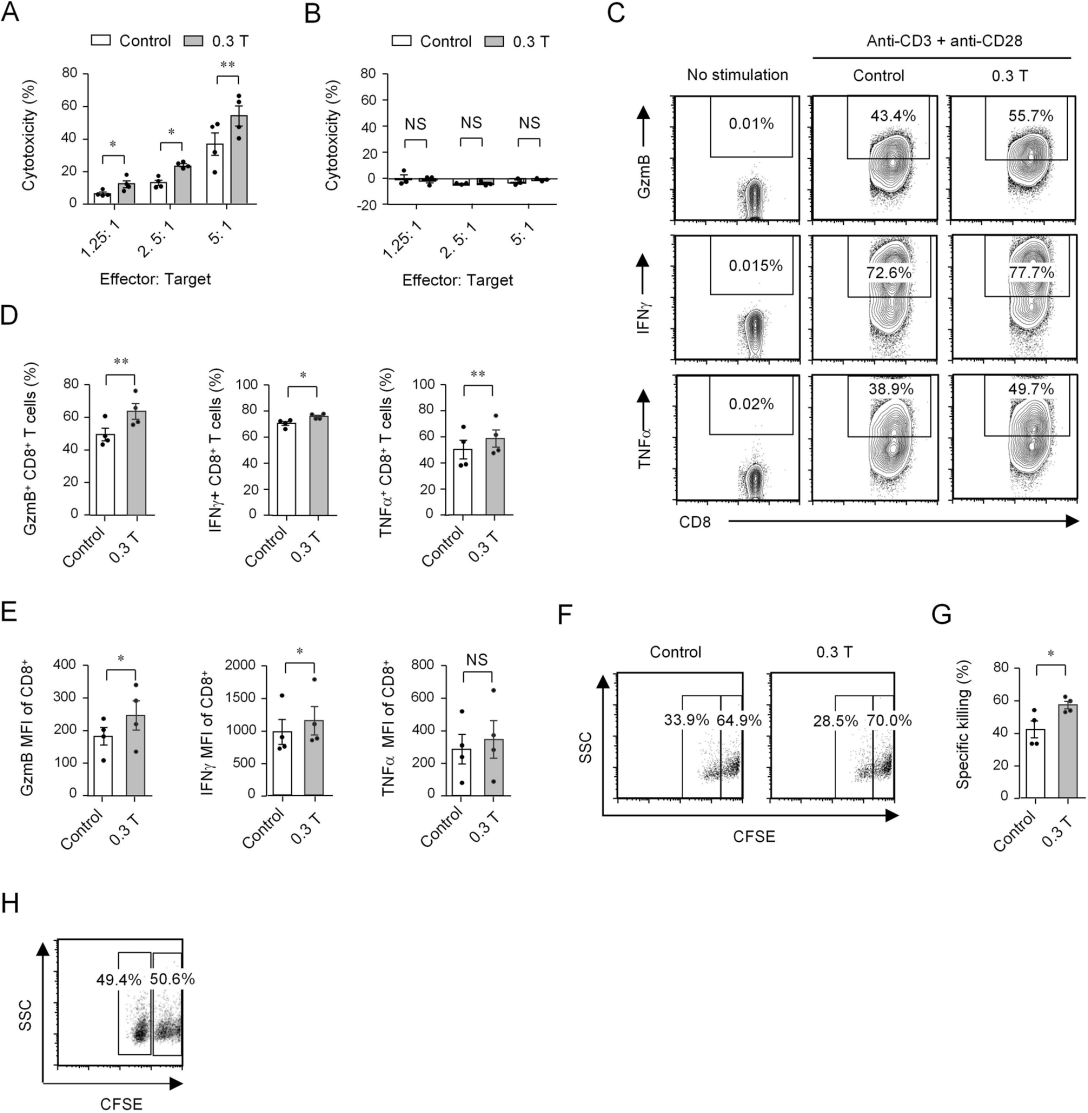
Moderate SMFs enhance CTLs cytotoxicity. (**A** and **B**) The cytotoxicity of CTLs generated from OT-I mice was assessed by LDH release with EL4 cells pulsing OVA (**A**, n = 4) or without pulsing OVA (**B**, n = 3). (**C**) Cytokine/granule production of CTLs generated from OT-I mice. Cell samples were stimulated with anti-CD3 and anti-CD28 antibodies in the presence of 0.3 T magnets, and control cells were treated without magnets. Cell samples with no stimulation were used to show the baseline of cytokine secretion. (**D** and **E**) Percentage (**D**) and MFI (**E**) statistics for the expression of GzmB, IFNγ and TNFα of CTLs generated from OT-I mice (n = 4). Cell samples were treated with 0.3 T magnets, and samples treated without magnets were used as controls. (**F**) In vivo cytotoxicity assay as indicated by the CFSE^low^ and CFSE^high^ populations analyzed by flow cytometry. SMF-treated and control CTLs (3 × 10^6^) were intravenously injected into recipient mice, followed by injection of OVA_257–264_-pulsed (CFSE^low^) and nonpulsed (CFSE^high^) splenocytes (5 × 10^6^) to measure in vivo cytotoxicity. (**G**) The killing percentage as revealed by the in vivo cytotoxicity assay (n = 4). (**H**) The negative control of in vivo cytotoxicity assay as indicated by the CFSE^low^ and CFSE^high^ populations analyzed by flow cytometry. OVA_257–264_-pulsed (CFSE^low^) and nonpulsed (CFSE^high^) splenocytes without getting CTLs were injected into recipient mice to measure in vivo cytotoxicity. The cytotoxicity was calculated as [1 − (% CFSE^low^)/(% CFSE^high^)] × 100%. Data were analyzed by Student’s t-test; NS, no significance, *p < 0.05; **p < 0.01. Error bars indicate the s.e.m.

CD8^+^ T cells are also known as CD8^+^ CTLs, which kill cancer cells or infected cells by releasing perforin, granzymes, and cytokines^4,5,25^. Because the aforementioned results revealed that 0.3 T SMF enhanced CD8^+^ T cell granule and cytokine secretion, we wanted to determine whether 0.3 T SMF can promote the cytotoxicity of CTLs. LDH-based cytotoxicity assays were performed using stimulated CD8^+^ T cells isolated from the spleens of OT-I TCR transgenic mice as effector cells, which are ovalbumin-specific CD8^+^ T cells^26^. In addition, OVA_257–264_ pulsed tumor EL-4 cells were used as target cells^27,28^. The results demonstrated that 0.3 T SMF-treated CTLs showed enhanced cytotoxicity compared with control cells at various effector to target ratios (Fig. 5A). Moreover, we also performed the control experiment where we co-cultured CTLs and EL4 cells without pulsing OVA. The results revealed that the cytotoxicity percentage is quite near zero at various effector to target ratios and there was no obvious difference between 0.3 T SMF-treated CTLs and control cells (Fig. 5B). In addition, 0.3 T SMF-treated CTLs presented higher granule and cytokine secretion than control cells (Fig. 5C–E). This suggested that 0.3 T SMF enhanced CD8^+ ^T cell cytotoxicity by promoting granule and cytokine secretion in vitro. Next, we used an in vivo killing assay^28^ to test whether SMF-treated CTLs also present the enhanced cytotoxicity effect in vivo. SMF-treated CTLs and control CTLs, which were obtained by stimulating CD8^+ ^T cells isolated from the spleens of OT-I TCR transgenic mice, were intravenously injected into recipient mice. Then OVA_257–264_-pulsed splenocytes (i.e., CFSE^low^) and nonpulsed splenocytes (i.e., CFSE^high^) were used as target cells and intravenously coinjected at a 1:1 ratio into recipient mice. Non-pulsed splenocytes were not lysed and used as internal controls. The cytotoxicity was calculated as [1 − (% CFSE^low^)/(% CFSE^high^)] × 100%^28^. Compared to the control CTLs, the SMF-treated CTLs killed more OVA_257–264_-pulsed splenocytes in vivo, and the flow cytometry data showed that 28.5% versus 33.9% CFSE^low^-target cells left (Fig. 5F). Correspondingly, the SMF-treated CTLs exhibited a superior killing efficiency compared with the control CTLs (Fig. 5G). In addition, we also performed the control experiment of received pulsed splenocytes without getting CTLs. The FACS result showed that the cell percentage of either CFSE^low^ (plused with OVA peptide) or CFSE^high^ (unplused with peptide) was approaching 50% (Fig. 5H) and the killing percentage was only 2.37%, which was quite near zero (Fig. 5H). These data suggest that SMF-treated CTLs also present the enhanced cytotoxicity effect in vivo.

### Moderate SMF exposure promotes the antitumor function of CD8^+^ T cells in vivo

The above results revealed that SMF exposure enhanced CD8^+^ T cell cytotoxicity by promoting granule and cytokine secretion. Because CD8^+^ T cells play important roles in antitumor immunity by releasing granules and cytokines, we next examined whether SMF exposure affects the antitumor function of CD8^+^ T cells in vivo. To do so, we used mammary epithelium-specific transgenic mice that express the polyomavirus middle T antigen (PyMT) and treated them with or without SMFs to assess antitumor function. Resin fiber plates embedded with 0.3 T or 0.6 T button magnets were used to expose mice to the SMFs (as shown in Supplementary Fig. S9A), and resin fiber plates without magnets were used as controls. The magnetic intensity of these button magnets was heterogeneous, as the force was much higher in the center area than at the edges. In general, the average surface magnetic intensity of magnetic plates embedded with 0.3 T button magnets was approximately 0.18 T, and the average surface magnetic intensity of magnetic plates embedded with 0.6 T button magnets was approximately 0.31 T.

**Figure 6:**
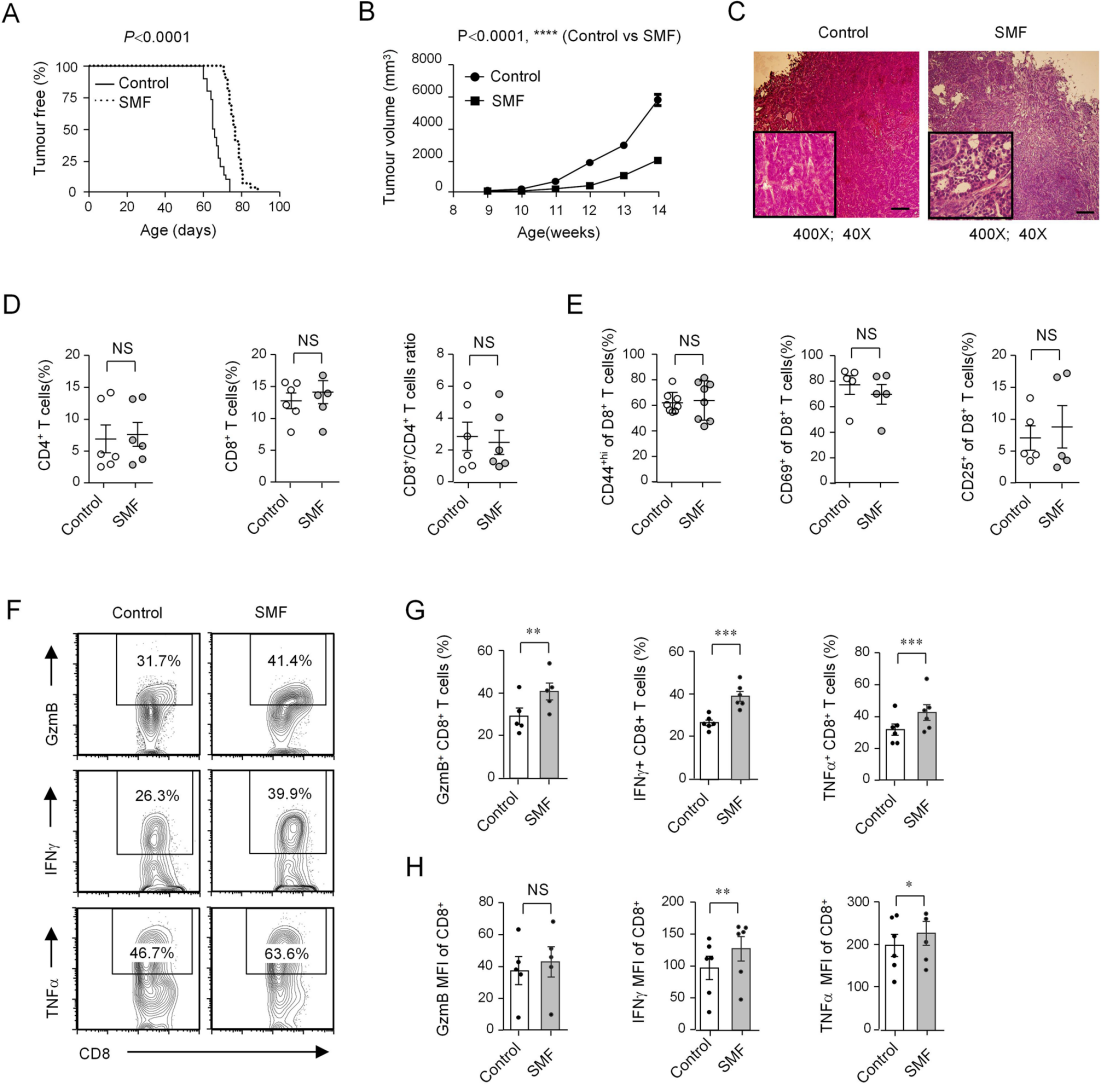
Moderate SMFs promote the antitumor response of CD8^+^ T cells in vivo. PyMT mice were exposed to 0.6 T magnetic plates, and littermates exposed to resin fiber plates without magnets were used as controls. Tumor onset (**A**; n = 30) and tumor growth (**B**; n = 17) of PyMT mice were constantly monitored. (**C**) Hematoxylin and eosin-stained mammary tumor sections from PyMT mice (magnification, × 40 and × 400; scale bars, 200 mm). (**D**) Percentage statistics for CD4^+^ T cells, CD8^+^ T cells, and the CD8^+^/CD4^+^ T cell ratio among tumor-infiltrating T cells in PyMT mice as analyzed by flow cytometry (n = 6). (**E**) Percentage statistics for the expression of CD69, CD44 and CD25 in tumor-infiltrating CD8^+^ T cells in PyMT mice as analyzed by flow cytometry (n = 5). (**F**) Cytokine/granule production of tumor-infiltrating CD8^+^ T cells in PyMT mice as analyzed by flow cytometry. (**G**, **H**) Percentage (**G**) and MFI (**H**) statistics for the expression of GzmB (n = 5), IFNγ (n = 6) and TNFα (n = 6) in tumor-infiltrating CD8^+^ T cells in PyMT mice as analyzed by flow cytometry. Data were analyzed by log-rank test (**A**), two-way ANOVA (**B**), or Student’s t-test (**D**, **E**, **G**) (NS, no significance, **p < 0.01; ***p < 0.001, ****p < 0.0001). Error bars indicate the s.e.m.

First, we observed the tumor onset time of magnetic plate-treated and control mice. The results showed that the time of tumor onset in the 0.3 T magnetic plate-treated mice was comparable to that in control mice (Supplementary Fig. S9B). In contrast, the time of tumor onset in the 0.6 T magnetic plate-treated mice was delayed compared to that in control mice (Fig. 6A). Then, we detected the tumor growth process by calculating the tumor volume. Consistent with the tumor onset, the mammary tumor growth showed no significant change between the 0.3 T magnetic plate-treated mice and control mice (Supplementary Fig. S9C). In contrast, the mammary tumor growth was reduced in 0.6 T magnetic plate-treated mice compared with that in control mice (Fig. 6B). In addition, histological analysis of the tumors showed that tumor development was significantly repressed in 0.6 T magnetic plate-treated mice, whereas invasive tumor growth was observed in control mice (Fig. 6C). These data suggested that mice exposed to 0.6 T magnetic plates exhibited apparent reductions in tumor growth.

Consequently, we examined whether SMFs influenced CD8^+^ T cell function in vivo by comparing 0.6 T magnetic plate-treated mice and control mice. First, we analyzed the tumor-infiltrating T cell subpopulation. The proportion of CD4^+^ T and CD8^+^ T cells and the CD8^+^/CD4^+^ T cell ratio were comparable between SMF-treated mice and control mice (Fig. 6D). Next, we examined the activation of tumor-infiltrating T cells. The proportions of CD44^+high^, CD69^+^ and CD25^+^ CD8^+^ T cells, which reflect the degree of activation, showed no obvious changes between SMF-treated mice and control mice (Fig. 6E). Moreover, the naïve population (CD62L^high^ CD44^low^) and effector memory population (CD62L^low^CD44^high^) of both CD4^+^ T and CD8^+^ T cells was also comparable (Supplementary Fig. S10A, B). The decreased tumor size detected in SMF-treated mice may have resulted from a reduction in Tregs (regulatory T cells) or MDSCs (myeloid-derived suppressor cells), which possess immunosuppressive activities. Our results showed that SMF-treated mice contained comparable proportions of tumor-infiltrating Treg cells and MDSCs as did the control mice (Supplementary Fig. S10C − F). Importantly, tumor-infiltrating CD8^+^ T cells in SMF-treated mice produced more antitumor granules and cytokines, including granzyme B, IFNγ and TNFα, than their counterparts from control mice (Fig. 6F,G). The MFI (mean fluorescence intensity) of granules and cytokines were also compared, and it showed that the expression of IFNγ and TNFα was elevated in SMF-treated mice (Fig. 6H). In contrast, the cytokine secretion levels from tumor-infiltrating CD4^+^ T cells were comparable between SMF-treated mice and control mice (Supplementary Fig. S11). Collectively, these data indicated that moderate SMF exposure uniquely potentiated the antitumor function of CD8^+^ T cells in vivo by promoting granule and cytokine secretion.

### Cancer immunotherapies using adoptive transfer of CTLs treated with SMFs

**Figure 7:**
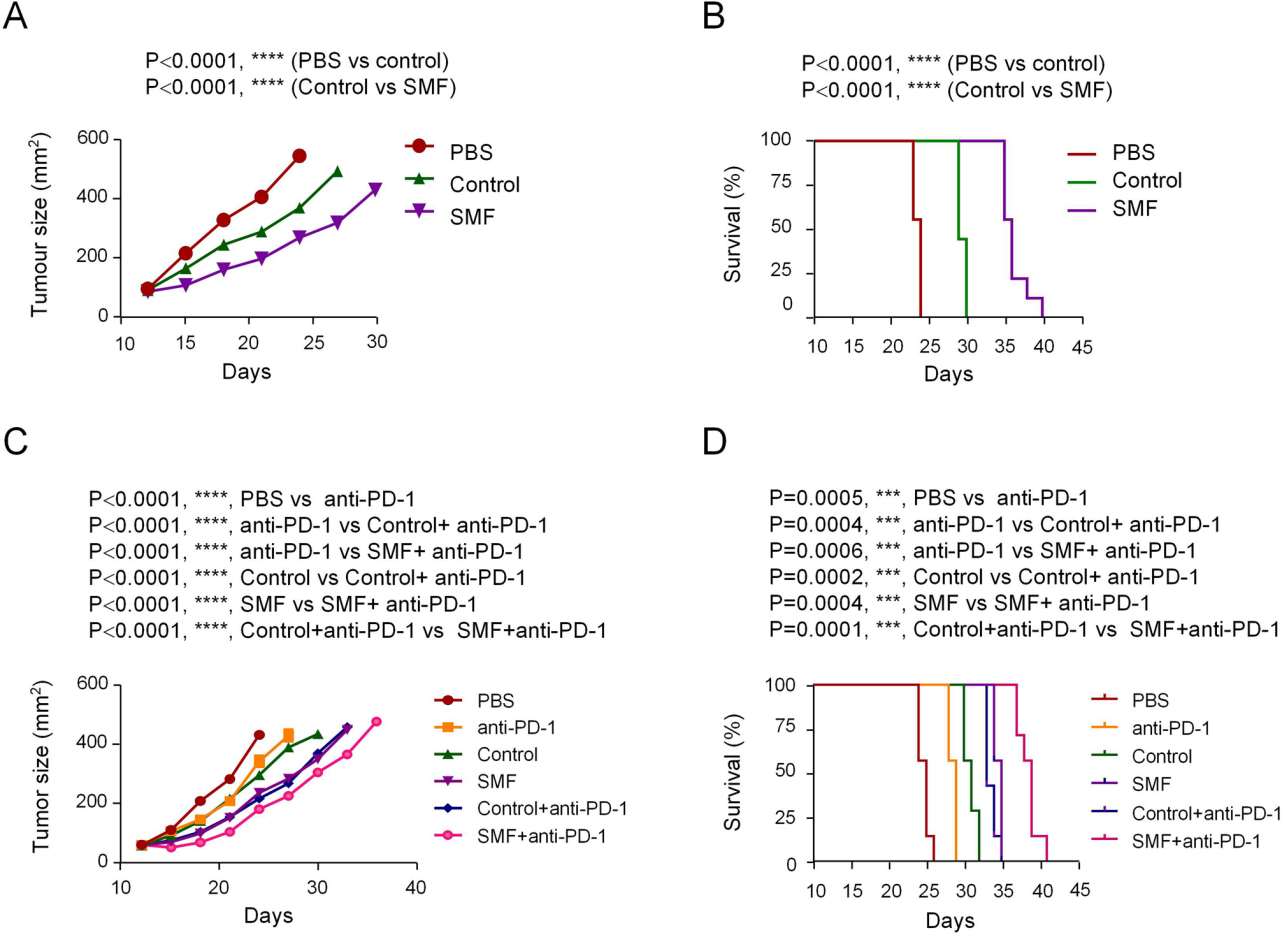
Cancer immunotherapies using adoptive transfer of CTLs subjected to moderate SMFs. Melanoma-bearing mice were treated with PBS, control CTLs and SMF-treated CTLs derived from OT-I TCR transgenic mice. Tumor size (**A**; n = 9) and survival (**B**; n = 9) were constantly monitored. (**C**, **D**) Effects of combined therapy (control CTLs and anti-PD-1; SMFs-treated CTLs and anti-PD-1) and monotherapies (anti-PD-1; control CTLs; SMFs-treated CTLs) in treating melanoma (n = 7). Data were analyzed by two-way ANOVA (**A**, **C**) or log-rank test (**B**, **D**) (***p < 0.001, ****p < 0.0001). Error bars indicate the s.e.m.

The above data demonstrated that moderate SMFs can enhance CD8^+^ T cell cytotoxicity both ex vivo and in vivo. Finally, we tested the antitumor function using the adoptive transfer of CTLs treated with 0.3 T SMF. We treated melanoma-bearing mice induced by B16F10-OVA_257–264_ cells with an intravenous injection of PBS, control CTLs or 0.3 T SMF-treated CTLs induced by stimulation of CD8^+^ T cells isolated from the spleens of OT-I TCR transgenic mice. Compared with mice injected with control CTLs, mice injected with SMF-treated CTLs showed stronger antitumor activity, which is evidenced by the smaller tumor size and prolonged survival time (Fig. 7A,B). Next, we further tested a combination therapy of SMF-treated CTLs and anti-PD-1 antibody, which showed a better efficacy than monotherapies in inhibiting tumor progression and extending survival. Furthermore, SMF-treated CTLs also showed stronger antitumor activity than did control CTLs when combined with anti-PD-1 antibody (Fig. 7C,D). These data suggested that adoptive transfer of SMF-treated CTLs exhibited enhanced antitumor effects, and the combined therapy of CTLs with anti-PD-1 antibody was more effective than either therapy alone.

## Discussion

In the present study, we examined the cellular effects of moderate SMFs on T cells and found that exposure to moderate SMFs enhanced granule and cytokine secretion from as well as ATP production in CD8^+^ T cells by promoting *Uqcrb* and *Ndufs6* genes of mitochondrial respiratory electron transport chain. Specially, the candidate magnetoreceptor genes *Isca1* and *Cry1*/*Cry2* involved in regulating the transcription level of *Uqcrb* and *Ndufs6*. We also observed that SMF exposure enhanced CD8^+^ T cell cytotoxicity and predominantly repressed tumor growth in vivo. In addition, the adoptive transfer of SMF-treated CTLs into tumor-bearing mice resulted in enhanced antitumor effects, and combining CTLs with anti-PD-1 antibody therapy was more effective. These results suggest that moderate SMFs can enhance CD8^+^ T cell cytotoxicity by promoting mitochondrial respiration via candidate magnetoreceptor genes and potentiate the antitumor function of CD8^+^ T cells.

In this study, we found that moderate SMFs enhanced ATP production and mitochondrial oxygen consumption of CD8^+^ T cells. Importantly, our study elucidated the specific molecular mechanism by which SMFs influence intracellular ATP levels—by upregulating *Uqcrb* and *Ndufs6* gene of mitochondrial respiratory electron transport chain. Uqcrb is one of the subunits of mitochondrial complex III, and Ndufs6 is a subunit of mitochondrial complex I, which play critical roles in the maintenance of mitochondrial complex, and mutation of *Uqcrb* or *Ndufs6* will lead to mitochondrial disorders^29,30^. Our study indicated that SMFs enhanced granule and cytokine secretion as well as ATP levels of CD8^+^ T cells by upregulating *Uqcrb* or *Ndufs6*, since their down expressions can inhibit the increased granule and cytokine secretion and elevated ATP levels. Specially, the mRNA level of *Uqcrb* is more predominantly upregulated compared with *Ndufs6* in the presence of magnetic field. This implicates Uqcrb as one of the subunits of mitochondrial complex III is more sensitive to magnetic field. It has been reported that mitochondrial oxygen consumption increased during T cell activation, and fueling mitochondria was sufficient to support T cell activation^31^. Moreover, mitochondrial complex III ROS production is essential for T cell activation and cytokine secretion. It’s possible the promoted cytokine secretion of CD8^+^ T cells in our study is mainly mediated by upregulation of Uqcrb of complex III and increased mitochondrial oxygen consumption^31^. In addition, it has been reported that mitochondrial respiration played important roles in IFNγ and IL-2 production in CD8^+^ T cells^32^. It showed that loss of MCJ, a negative regulator of mitochondrial respiration in CD8^+^ T cells, led to increased ATP production and mitochondrial oxygen consumption as well as cytokine secretion of CD8^+^ T cells^32^. These also drop us a hint that the enhanced cytokine secretion of SMF-treated CD8^+^ T cells in our study was caused by elevated ATP production and mitochondrial respiration.

Our study also revealed that SMFs promoted the transcription level of *Uqcrb* and *Ndufs6* via candidate magnetoreceptor genes *Isca1* and *Cry1*/*Cry2*. Knocking down *Isca1* or *Cry1*/*Cry2* led to reduced transcription of *Uqcrb* and *Ndufs6*, and can also prevent the increased granule and cytokine secretion as well as ATP production of SMF treated CD8^+^ T cells, and *Isca1* had more predominant effects. *Isca1* and *Cry1*/*Cry2* have been identified as candidate magnetoreceptor genes^9,11,20,33^. but their downstream targets and pathways are largely unknown. It’s probable that the enhanced cytokine secretion of SMF-treated CD8^+^ T cells in our study was caused by upregulation of mitochondrial respiratory genes *Uqcrb* and *Ndufs6*, which were mediated by candidate magnetoreceptor genes *Isca1* and *Cry1*/*Cry2*. Our study established a novel link between magnetoreceptor genes and mitochondrial respiratory genes, which will be valuable for understanding the mechanism how cells receive magnetic signals and transduce into biological signals and cause biological effects. In addition, the unique relationship among SMFs, mitochondrial metabolism and CD8^+^ T cell cytotoxicity provides a novel perspective for understanding the immunophysiological effects of SMFs as well as the mechanism of CD8^+^ T cell cytotoxicity.

In recent years, many efforts have been made to enhance the antitumor immunity of T cells. For example, strategies involving the transfer of ex vivo expanded T cells to patients have rapidly advanced with the emergence of TCR and chimeric antigen receptor (CAR)-T cell therapies, both of which can redirect T cells to more efficiently target cancer antigens^34,35^. These are both cellular engineering approaches that use genome editing techniques to insert large transgenes into target cells^36^. Moreover, anti-PD-1 therapy has also achieved great success by selectively restoring tumor-induced immune deficiencies in the tumor microenvironment^37^. Although CAR-T cell therapy and anti-PD-1 therapy have shown remarkable success in treating hematological cancers but are much less effective against solid tumors^38,39^, exploring novel methods and strategies that can enhance the antitumor functions of T cells are still necessary. In this study, we found that SMF exposure enhanced the antitumor function of CD8^+^ T cells in vivo, and the adoptive transfer of SMF-treated CTLs resulted in enhanced antitumor effects. This provides a new perspective of promoting the antitumor function of CD8^+^ T cells using a physical and non-invasive method, which is simpler and less expensive than existing methods.

In recent years, the cellular effects of SMFs have been widely studied^12–16^. Various experimental parameters, such as cell types, magnetic field parameters (including magnetic field intensity and direction), and exposure time, often led to different cellular effects. In our study, we screened the cellular effects of moderate SMFs on the function of both CD4^+^ T and CD8^+^ T cells at various time points after TCR stimulation and upon treatment with SMFs at different intensities. We observed that exposure to moderate SMFs enhanced granule and cytokine secretion of CD8^+^ T cells at 72 h after TCR stimulation but showed no obvious effects at 24 h and 48 h after stimulation. Moreover, SMFs did not affect cytokine secretion of CD4^+^ T cells at any of the time points. In addition, our in vivo studies also revealed that exposure to moderate SMFs promoted granule and cytokine secretion from tumor-infiltrating CD8^+^ T cells from PyMT mice but did not affect cytokine secretion from CD4^+^ T cells. These results suggested that SMFs showed highly variable effects between CD4^+^ T cells and CD8^+^ T cells. Considering the diversify and plasticity of the immune system, the biological effects of SMFs on different subsets of immune cells with various intensities or stimulation times are still worth to be further systematically studied.

## Materials and methods

### Mice

C57BL/6 mice were purchased from SLAC. OT-I TCR transgenic mice and PyMT transgenic mice were obtained from The Jackson Laboratory. All mice were maintained in a specific pathogen-free facility and were genotyped by PCR. All animal experiments were approved by the Institutional Animal Care and Use Committee of the Shanghai Institute of Biochemistry and Cell Biology (Chinese Academy of Sciences). We confirmed that all experiments were performed in accordance with relevant guidelines and regulations.

### Cell lines

B16-F10-OVA and EL-4 cell lines were kindly provided by Prof. Chenqi Xu (Chinese Academy of Sciences). B16-F10 cells were cultured in Dulbecco’s modified Eagle’s medium supplemented with 10% fetal bovine serum, l-glutamine (2 mM), penicillin (100 U/ml), and streptomycin (10 U/ml). EL-4 cells were cultured in RPMI 1,640 medium supplemented with 10% fetal bovine serum, l-glutamine (2 mM), penicillin (100 U/ml), and streptomycin (10 U/ml).

### Permanent magnets and magnetic plates

Permanent magnets with an intensity of 0.3 T and 0.6 T were purchased from the Hangzhou Permanent Magnet Group Co., Ltd. The size of each 0.3 T permanent magnet is 10 cm × 10 cm × 5 cm, and the size of the 0.6 T permanent magnets is 10 cm × 10 cm × 10 cm. The magnetic intensity of the permanent magnets slowly attenuates with a distance of approximately 1 mm corresponding to a decrease in 5 mT. The resin fiber plates embedded with or without 0.3 T or 0.6 T button neodymium magnets were provided by Heye Health Technology Co., Ltd. of Zhejiang Province. The diameter of each button magnet is 1 cm, and the distance between any two button magnets is 2.5 cm. The magnetic intensity of these button magnets rapidly attenuates with a distance of approximately 1 mm corresponding to a decrease in 50 mT. The size of these resin fiber plates (32 cm × 12.5 cm × 1.5 cm) were designed to fit the mouse cages and were placed at the bottom of the cages. All the permanent magnets and magnetic plates were placed with the N pole up and the S pole down. The magnetic field strength was measured by KANETEC TESLA METER MODEL TM-801.

### Antibodies and reagents

Anti-CD4 (RM4-5), anti-CD8a (53-6.7), anti-CD44 (IM-7), anti-CD62L (MEL-14), anti-CD69 (H1.2F3), anti-Ly6G (Gr1) (RB6-8C5), anti-CD11b (M1/70), anti-TNFα (MP6-XT22), anti-IL-2 (JES6-5H4), and anti-IL-4 were purchased from BD Pharmingen. Anti-mFoxp3 (FJK-16 s), anti-granzyme B (NGZB) and anti-IL-17 (eBio17B7) were purchased from eBioscience. Anti-CD25 (PC61), anti-IFNγ (XMG1.2), and FITC Annexin V reagents were purchased from Biolegend. Anti-CD3 (145-2C11) and anti-CD28 (37.51) antibodies used for in vitro stimulation were obtained from BD Pharmingen. Brefeldin A was obtained from eBioscience, PMA and ionomycin were obtained from Merck. Collagenase IV used for tumor tissue digestion was purchased from Sigma.

### T cell isolation and staining

CD4^+^ T cells or CD8^+^ T cells were isolated from the splenocytes of C57BL/6 mice using a CD4^+^ or CD8^+^ T cell negative selection kit (Stem Cell) and were stimulated with 1 μg ml^−1^ plate-bound anti-CD3 and anti-CD28 antibodies using 48-well cell culture plates. On the first day of stimulation, cell samples were seeded at 7 × 10^5 ^cells per well, then subculture at 1:2 ratio at 48 h after stimulation. Cell culture plates were placed on magnets once stimulated, and control cells were treated in the absence of magnets. To analyze the tumor-infiltrating T cells, tumors were first digested with collagenase IV, and tumor-infiltrating leukocytes were isolated by 40–80% (v/v) Percoll (GE) gradient centrifugation as previously described^40^. To measure the cytokine production of CD4^+^ or CD8^+^ T cells, the isolated cells were restimulated with PMA (50 ng ml^−1^) plus ionomycin (1 μM) for 4 h in the presence of 5 μg ml^−1^ brefeldin A and then stained with antibodies. Intracellular staining was performed after 10 min of fixation (2% formaldehyde in PBS) at room temperature and 5 min of permeabilization in IC staining buffer (0.1% saponin, 0.1% bovine serum albumin Hank’s balanced salt solution) at 4 °C. Unstimulated T cells or T cells stained with isotype control antibody were used as negative controls. Cell fluorescence was determined using a two-laser FACSCalibur (BD Biosciences) flow cytometer, and data were analyzed with FlowJo software (TreeStar, Inc., Olten, Switzerland).

## Measurement of CD8^+^ T cell cytotoxicity in vitro

A CD8^+^ T cell cytotoxicity assay was performed as previously described^41^. Purified CD8^+^ T cells from OT-I mice were stimulated with anti-CD3/anti-CD28 mAbs in vitro for 4 days (96 h) as effector CTL cells, which were treated with or without 0.3 T magnets. EL-4 cells were used as target cells, which were pulsed with 10 nM OVA_257–264_ for 30 min. After EL-4 cells and CTLs were each washed three times with PBS, they (1 × 10^5^ for both cell types) were mixed in killing medium (phenol-free RPMI 1,640, 2% FBS) at respective ratios of 1.25:1, 2.5:1 and 5:1. After 6 h, the cytotoxic efficiency was measured using a CytoTox 96 Non-Radioactive Cytotoxicity kit (Promega) which quantified the release of endogenous lactate dehydrogenase (LDH) from EL-4 cells.

### Measurement of CD8^+^ T cell cytotoxicity in vivo

The in vivo killing assay was performed as previously described with some modifications^28^. CD8^+^ CTL cells treated with or without 0.3 T magnets were induced from OT-I mice as previously mentioned. Then, CTLs (3 × 10^6^) were intravenously injected into recipient C57BL/6 mice. After 4 h, CFSE^low^ (1 μM)-labeled splenocytes were pulsed with 10 nM OVA_257–264_ peptide, mixed at a 1:1 ratio with unpulsed CFSE^high^ (10 μM)-labeled splenocytes (5 × 10^6^) and intravenously injected into recipient mice. The OVA_257–264_-pulsed splenocytes (CFSE^low^) and nonpulsed splenocytes (CFSE^high^) were used as target cells. Non-pulsed splenocytes were not lysed and used as internal controls. After 6 h, splenocytes from the recipient mice were analyzed by flow cytometry to detect CFSE expression, and cytotoxicity was calculated as [1 − (% CFSE^low^)/(% CFSE^high^)] × 100%.

### Treatment of melanoma by adoptive SMF-treated T cell transfer

Adoptive T cell transfer was performed as previously described^27^. B16F10-OVA cells (2 × 10^5^) were subcutaneously injected into 8- to 10-week old C57BL/6 mice. On day 12, melanoma-bearing mice with similar tumor sizes were randomly divided into three groups (*n* = 7–10) and intravenously injected with PBS, CTLs (1.5 × 10^6^) generated from OT-I mice treated with 0.3 T magnets or control CTLs (1.5 × 10^6^) treated without magnets. For combined therapy of CTLs and anti-PD-1 antibody, another three groups were formed: anti-PD-1 antibody, CTLs and anti-PD-1 antibody in the presence of 0.3 T magnets, and CTLs and anti-PD-1 antibody in the absence of the magnets. Anti-PD-1 antibody (RMP1-14, Bio X Cell, 200 μg per injection) was intraperitoneally injected every 3 days. Starting on day 12, the tumor size was measured every 3 days, and the animal survival rate was recorded daily. Tumor size was calculated as length × width. Mice with tumor sizes larger than 20 mm on the longest axis were euthanized for ethical purposes.

### PyMT tumor development and histology staining

PyMT mice (FYB background) were treated with magnetic plates or control plates since 3 weeks old and examined by visual inspection and palpation every 2–3 days to monitor tumor onset. The tumor size was measured weekly with digital Vernier calipers, and the tumor volume was estimated using the formula A × B^2^/2, where A is the sagittal length (mm) and B is the cross length (mm). Mammary tumors from sacrificed mice were dissected, fixed in 4% (v/v) buffered neutral formalin and embedded in paraffin. Five-micron tissue sections were stained with hematoxylin and eosin. All images were observed under an Olympus BX51 microscope and acquired with a DP71 camera (Olympus).

### RNA-Seq, library generation and bioinformatics analysis

RNA-Seq, library generation and bioinformatics analysis were performed as previously described^42^. RNA was extracted, purified and checked for integrity using an Agilent Bioanalyzer 2100 (Agilent Technologies). Libraries were generated for sequencing using a SMARTer Stranded Total RNA-Seq Kit-Pico Input Mammalian (Illumina). Libraries were sequenced using an Illumina HiSeq X Ten sequencer. Only genes with a fold change ≥ 2 and a *p* value ≤ 0.05 were defined as differentially expressed. Gene Ontology biological processes that were enriched based on the differentially regulated genes were identified by DAVID (modified Fisher exact *p* value < 0.05)^43^.

### Real-time reverse transcriptase-PCR

Total RNA from cell samples was extracted with TRIzol (Invitrogen), reverse-transcribed using a SuperScript III First-Strand kit (Invitrogen) and subjected to quantitative reverse transcription PCR (qRT-PCR). The gene-specific primers were designed based on mRNA sequences retrieved from NCBI. The NCBI reference sequence numbers and primer sequences (5′–3′) of the genes are provided as follows:

- *Ndufc1* (NM_025523.1) (forward, CACGGTCGAAGTTCTATGTC; reverse, TTGTGTGTTTGGATGAGATAAATC);
- *Atp6v0b* (NM_033617.3) (forward, AGTTGCTCTACCTCGGGATCT; reverse, ATGCCACATCAAAGCGAAAGC);
- *Idh3b* (NM_130884.4) (forward, AGGCACAAGATGTGAGGGTG; reverse, CAGCAGCCTTGAACACTTCC);
- *Ndufs6* (NM_010888.2) (forward, GGGGAAAAGATCACGCATACC; reverse, CAAAACGAACCCTCCTGTAGTC);
- *Uqcrc2* (NM_025899.2) (forward, AAAGTTGCCCCGAAGGTTAAA; reverse, GAGCATAGTTTTCCAGAGAAGCA);
- *Uqcrb* (NM_026219.2) (forward, GGCCGATCTGCTGTTTCAG; reverse, CATCTCGCATTAACCCCAGTT);
- *Ndufa13* (NM_001382215.1) (forward, ACGGCCCCATCGACTACAA; reverse, CCTGGTTCCACCTCATCATTCT);
- *Atp5k* (NM_007507.3) (forward, GTTCAGGTCTCTCCACTCATCA; reverse, CGGGGTTTTAGGTAACTGTAGC);
- *Uqcrfs1* (NM_025710.2) (forward, GAGCCACCTGTTCTGGATGTG; reverse, GCACGACGATAGTCAGAGAAGTC);
- *Ndufa10* (XM_030242321.1) (forward, ACCTTTCACTACCTGCGGATG; reverse, GTACCCAGGGGCATACTTGC);
- *Gzmb* (NM_013542.3) (forward, TCTCGACCCTACATGGCCTTA; reverse, TCCTGTTCTTTGATGTTGTGGG);
- *Tnf* (NM_001001495) (forward, CTGGATGTCAATCAACAATGGGA; reverse, ACTAGGGTGTGAGTGTTTTCTGT);
- *Ifng* (NM_008338.4) (forward, TCCTCGCCAGACTCGTTTTC; reverse, GTCTTGGGTCATTGCTGGAAG);
- *Isca1* (NM_026921.4) (forward, CTTAAAGACAAACCTGAGCAT; reverse, TTCCACATAGTCCATCTCTG);
- *Isca2* (NM_028863.1) (forward, AACAACATCTTCCATTCCAGAG; reverse, CCCTTCGGTGATTTCCAGAA);
- *Cry1* (NM_007771.3) (forward, TTGAAGAGTTACTGCTTGATG; reverse, ACGCCTAATATAGTCTCCATT);
- *Cry2* (NM_009963.4) (forward, TGTGGGCATCAACCGATGG; reverse,
- CGGACTACAAACAGACGCGAA); and.
- *Beta-actin* (XM_030254057.1) (forward, GACGGCCAGGTCATCACTATTG; reverse, AGGAAGGCTGGAAAAGAGCC).

### Construction of knockdown primary T cells

For the construction of knockdown T cells, Plat-E cells were transfected with MLP shRNA plasmids, and retrovirus supernatants were harvested 48 h after transfection. Purified CD8^+^ T cells from C57BL/6 mice were stimulated with plate-bound anti-CD3 and anti-CD28 antibodies for 24 h, incubated in media containing retrovirus supernatants and centrifuged at 1,500 g for 2 h at 32 °C. Retroviral supernatants were then replaced by fresh culture medium, and cells were stimulated with plate-bound anti-CD3 and anti-CD28 antibodies for 72 h in the presence or absence of 0.3 T magnets. GFP-positive cells were sorted and subjected to real-time reverse transcriptase-PCR to test the knockdown efficiency. In order to rule out an off-target effect, we designed at least three shRNAs per gene and only the shRNA having predominant knockdown efficiency was finally used. In addition, the cell samples transfected with empty vector was used as negative controls^44^.

- Mouse *Uqcrb*-target shRNA: 5′-TGCTGTTGACAGTGAGCGCAAGAAGTGATCTTTTAGTTAATAGTGAAGCCACAGATGTATTAACTAAAAGATCACTTCTTTTGCCTACTGCCTCGGA-3′. Mouse *Ndufs6*-target shRNA: 5′-TGCTGTTGACAGTGAGCGCGCCATTGATTTGATAGCACAATAGTGAAGCCACAGATGTATTGTGCTATCAAATCAATGGCATGCCTACTGCCTCGGA-3′.
- Mouse *Isca1*-target shRNA: 5′-TGCTGTTGACAGTGAGCGATGCCTCGTGGGTGAAAATAAATAGTGAAGCCACAGATGTATTTATTTTCACCCACGAGGCAGTGCCTACTGCCTCGGA-3′.
- Mouse *Cry1*-target shRNA:
- 5′-TGCTGTTGACAGTGAGCGCCCGCCTCTTTATTTACATCTATAGTGAAGCCACAGATGTATAGATGTAAATAAAGAGGCGGATGCCTACTGCCTCGGA-3′.
- Mouse *Cry2*-target shRNA:
- 5′-TGCTGTTGACAGTGAGCGCCAGTTTGTTTGTGAATATTTATAGTGAAGCCACAGATGTATAAATATTCACAAACAAACTGTTGCCTACTGCCTCGGA-3′.

### Intracellular ATP concentration

The intracellular ATP concentration in CD8^+^ T cells (10^5^) was detected using an ATP Assay Kit (Beyotime) following the manufacturer’s recommendations. The luminescence of each sample was measured using a GLOMAX 20/20 Luminometer.

### Mitochondrial respiration

Using the Seahorse MitoStress Test Kit, we analyzed the OCRs and ECARs of CD8^+^ T cells under basal conditions and in response to 1 μM oligomycin, 1 μM fluorocarbonyl cyanide phenylhydrazone (FCCP), 1 μM rotenone and 1 μM antimycin, 10 mM glucose and 100 mM 2-Deoxy-D-glucose (2-DG). The link between OCRs and mitochondrial ATP production (ATP-linked OCR) was determined by subtracting the OCR of CD8^+^ cells reached after treatment with oligomycin from the OCR obtained at baseline. All extracellular flux analyses were performed using an XF-24 Extracellular Flux Analyzer (Seahorse Bioscience) as recommended by the manufacturer.

### Statistical analysis

Statistical analyses were performed with GraphPad Prism 6. Data are expressed as the mean ± SEM, and a two-tailed paired or unpaired Student’s *t*-test was used, unless otherwise indicated, to determine statistical significance. Data are representative of or combined from at least three independent experiments. For all experiments: *p < 0.05; **p < 0.001; ***p < 0.0001, ****p < 0.0001.

## Supplementary information

Supplementary Information.

## Data Availability

The authors declare that the data supporting the findings of this study are available within the article and its supplementary information files or from the corresponding author upon request. All sequencing data that support the findings of this study have been deposited in the Gene Expression Omnibus (GEO) of the National Center for Biotechnology Information (NCBI) under accession number GSE113858 (reviewer access link: https://www.ncbi.nlm.nih.gov/geo/query/acc.cgi?acc=GSE113858, token: orqlmoqkfbwpxgp).
