# Supplementary material for: Moderate static magnetic fields enhance antitumor CD8+ T cell function by promoting mitochondrial respiration
Source: Sci Rep. 2020 Sep 3;10:14519. doi: 10.1038/s41598-020-71566-x (PMC7471296; doi:10.1038/s41598-020-71566-x)
Supplement: Supplementary file 1 — Supplementary Information. [file 41598_2020_71566_MOESM1_ESM.pdf]

Supplementary information

Moderate static magnetic fields enhance antitumor CD8+ T cell function by promoting mitochondrial respiration

Xiaoyan Zhu[1]*, Yan Liu[1,2], Xianxia Cao[1,2], Haifeng Liu[1], Ao Sun[1], Hao Shen[1], Jingyao Zhao[1], Ronghong Li[1], Ligang Wu[1], Zhicai Fang[3], Hui Wang[4], and Qiwei Zhai[4].

[1]State Key Laboratory of Cell Biology, CAS Center for Excellence in Molecular Cell Science, Shanghai Institute of Biochemistry and Cell Biology, Chinese Academy of Sciences, University of Chinese Academy of Sciences, China.

[2]School of Life Science and Technology, Shanghai Tech University, Shanghai, 200031, China

[3]Heye Health Technology Co., Ltd., Zhejiang, China.

[4]Key Laboratory of Nutrition and Metabolism, CAS Center for Excellence in Molecular Cell Sciences, Institute for Nutritional Sciences, Shanghai Institutes for Biological Sciences, Chinese Academy of Sciences, University of Chinese Academy of Sciences, China.

*Correspondence and requests for materials should be addressed to X.Y. (xyzhu01@sibcb.ac.cn)

Keywords: moderate SMFs, CD8+ T cells, cytotoxicity, mitochondrial metabolism, antitumor function

Supplementary information

Figure. S1. The permanent magnets used in the study.
Figure. S2. Cellular effects of moderate SMFs on the expression of $CD4^+$ T cell activation markers.
Figure. S3. Cellular effects of moderate SMFs on the expression of $CD8^+$ T cell activation markers.
Figure. S4. Moderate SMFs have no obvious effects on $CD4^+$ T cell cytokine secretion.
Figure S5. Moderate SMFs have no obvious effects of $CD8^+$ T cell granule and cytokine secretion at 24h and 48h stimulation.
Figure. S6. Sham materials have no obvious effects on $CD8^+$ T cell granule and cytokine secretion.
Figure. S7. Moderate SMFs do not influence $CD8^+$ T cell proliferation and apoptosis.
Figure. S8. Moderate SMFs promote gene expressions related with mitochondrial respiratory electron transport chain.
Figure. S9. The magnetic plates embedded with 0.3 T magnets have no obvious effects on tumor on set and growth.
Figure. S10. Moderate SMFs do not affect subpopulations of tumour-infiltrating $CD8^+$ T cells.
Figure. S11. Moderate SMFs do not influence cell cytokine secretion of tumour-infiltrating $CD4^+$ T cells.

Supplementary Figure 1.

A B

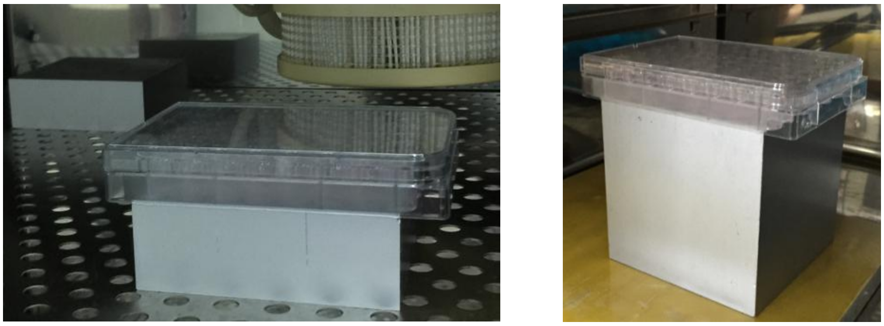

Figure S1. The permanent magnets used in the study.
The 48-well cell culture plates with cell samples are placed on 0.3 T magnet (**A**) and 0.6 T magnet (**B**).

Supplementary Figure 2.

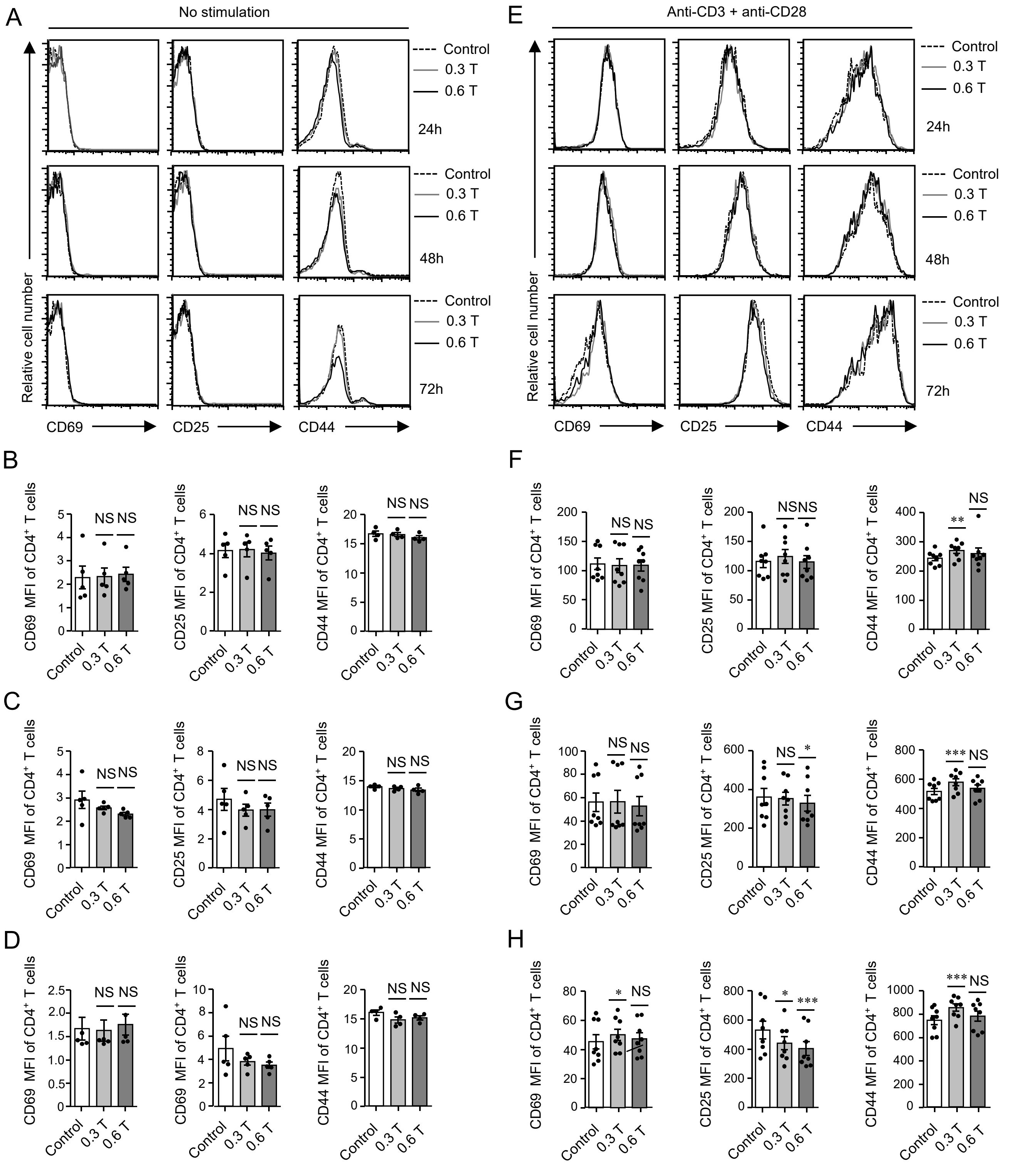

Figure S2. Cellular effects of moderate SMFs on the expression of CD4^{+} T cell activation markers.
(**A**) Histograms showing the surface expression of CD69,CD25 and CD44 of CD4^{+} T cells analyzed by flow cytometry. Cells were cultured with no stimulation for 24h, 48h and 72h. (**B, C and D**) MFI (mean fluorescence intensity) statistics for expression of CD69, CD25 and CD44 of CD4^{+} T cells with no stimulation for 24h (**B**), 48h (**C**), and 72h (**D**) (n=4-5). (**E**) Histograms showing the surface expression of CD69,CD25 and CD44 of CD4^{+} T cells analyzed by flow cytometry. Cells were stimulated with anti-CD3 and anti-CD28 for 24h, 48h and 72h. (**F, G and H**) MFI statistics for expression of CD69, CD25 and CD44 of CD4^{+} T cells stimulated with anti-CD3 and anti-CD28 antibodies for 24h (**F**), 48h (**G**), and 72h (**H**) (n=8). Cell samples were cultured in the presence of 0.3 T or 0.6 T permanent magnets, and control cells were treated without magnets. Data were analyzed by Student's t-test; NS, no significance, *$P < 0.05$, **$P < 0.01$, ***$P < 0.001$. Error bars indicate the s.e.m. Data are representative of or combined from at least three independent experiments.

Supplementary Figure 3.

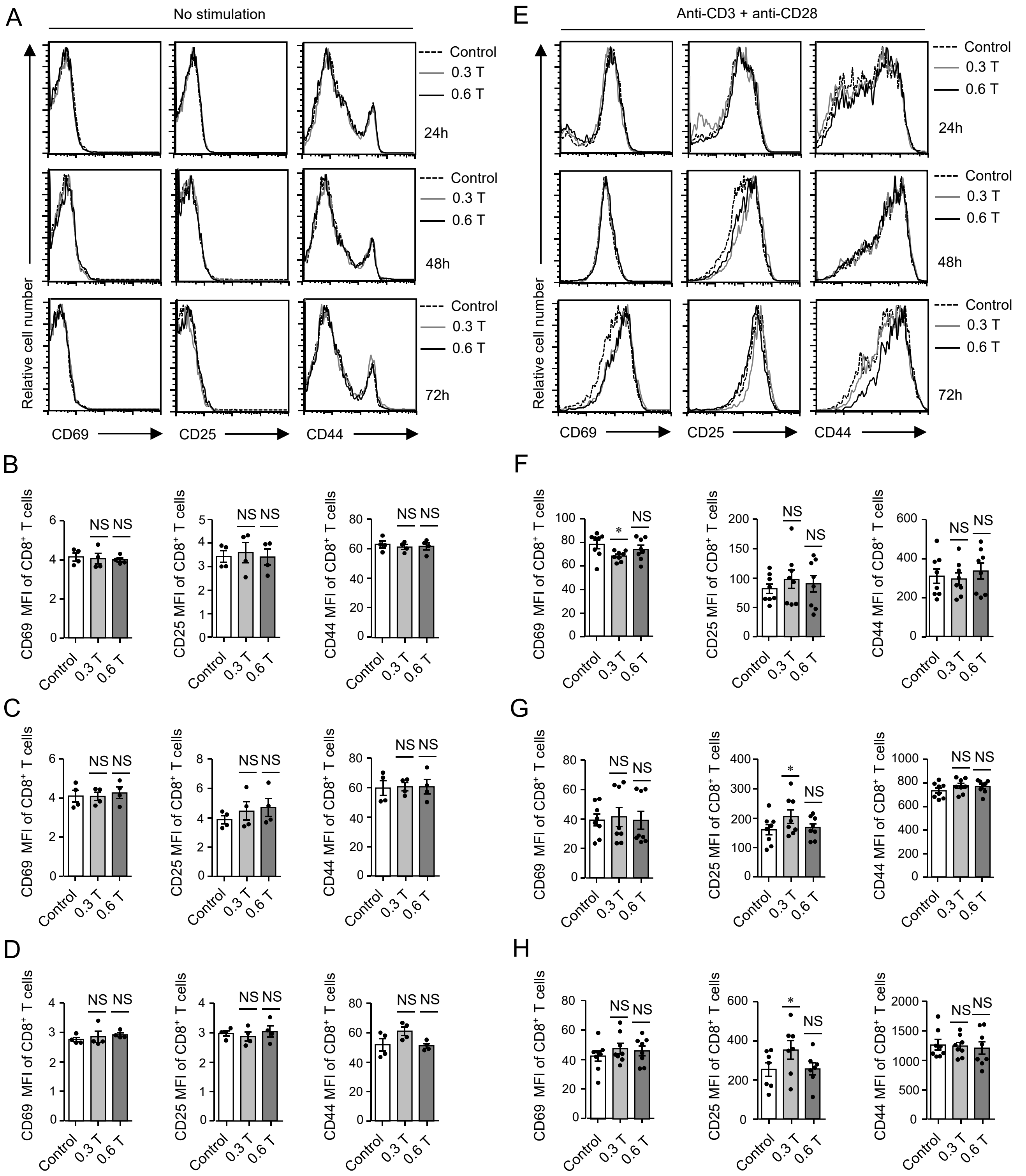

Figure S3. Cellular effects of moderate SMFs on the expression of CD8+ T cell activation markers. (**A**) Histograms showing the surface expression of CD69,CD25 and CD44 of CD8+ T cells analyzed by flow cytometry. Cells were cultured with no stimulation for 24h, 48h and 72h. (**B, C and D**) MFI (mean fluorescence intensity) statistics for expression of CD69, CD25 and CD44 of CD8+ T cells with no stimulation for 24h (**B**), 48h (**C**), and 72h (**D**) (n=4). (**E**) Histograms showing the surface expression of CD69,CD25 and CD44 of CD8+ T cells analyzed by flow cytometry. Cells were stimulated with anti-CD3 and anti-CD28 for 24h, 48h and 72h. (**F, G and H**) MFI statistics for expression of CD69, CD25 and CD44 of CD8+ T cells stimulated with anti-CD3 and anti-CD28 antibodies for 24h (**F**), 48h (**G**), and 72h (**H**) (n=8). Cell samples were cultured in the presence of 0.3 T or 0.6 T permanent magnets, and control cells were treated without magnets. Data were analyzed by Student's t-test; NS, no significance, $*P < 0.05$. Error bars indicate the s.e.m. Data are representative of or combined from at least three independent experiments.

Supplementary Figure 4.

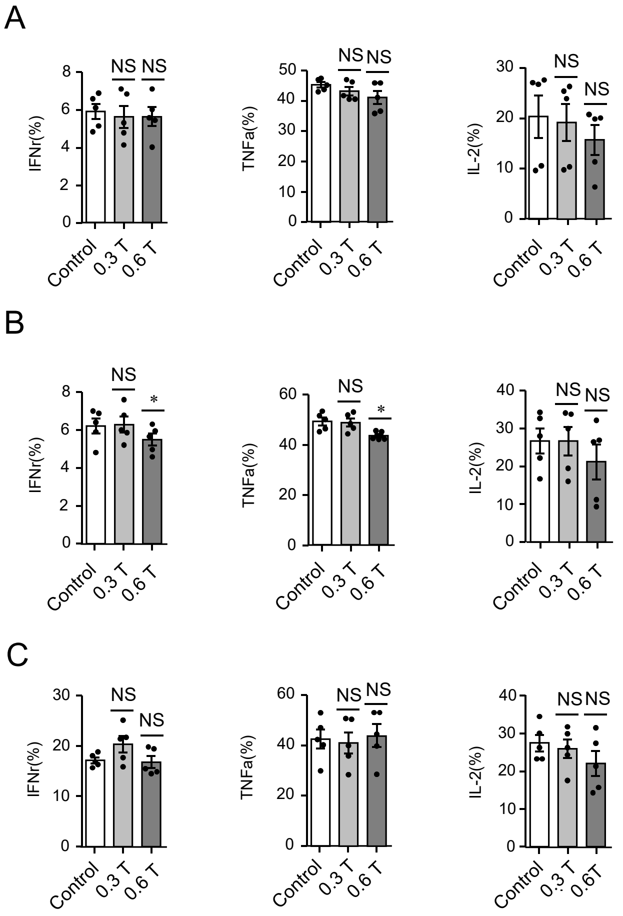

Figure S4. Moderate SMFs have no obvious effects on $CD4^+$ T cell cytokine secretion. Percentage statistics for the expression of IFNγ, TNFα and Il-2 of purified $CD4^+$ T cells stimulated with anti-CD3 and anti-CD28 antibodies for 24h (**A**), 48h (**B**), and 72h (**C**) (n=5). Cell samples were stimulated in the presence of 0.3 T or 0.6 T permanent magnets, and control cells were treated without magnets. Data were analyzed by Student's t-test; NS, no significance, *$P < 0.05$. Error bars indicate the s.e.m. Data are representative of or combined from at least three independent experiments.

Supplementary Figure 5.

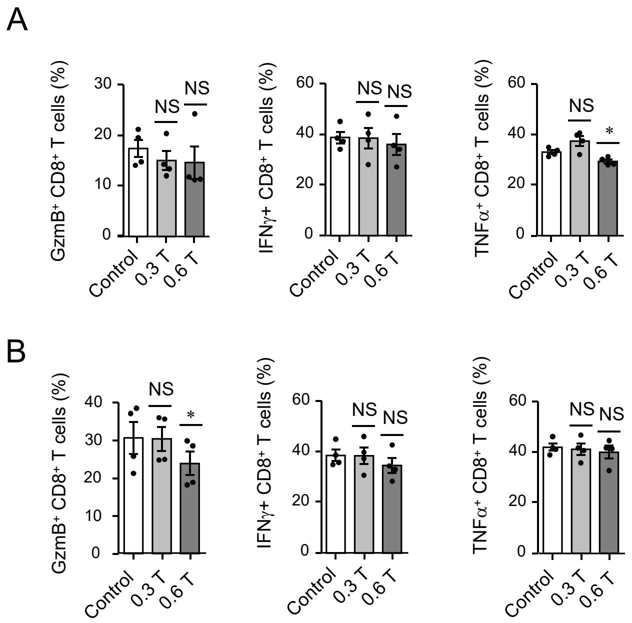

Figure S5. Moderate SMFs have on obvious effects of CD8^{+} T cell granule and cytokine secretion at 24h and 48h stimulation.

Percentage statistics for the expression of GzmB, IFNγ and TNFα of CD8^{+} T cells stimulated for 24h (A, n=4) and 48h (B, n=4). Data were analyzed by Student's t-test; NS, no significance, *P < 0.05. Error bars indicate the s.e.m. Data are representative of or combined from at least three independent experiments.

Supplementary Figure 6.

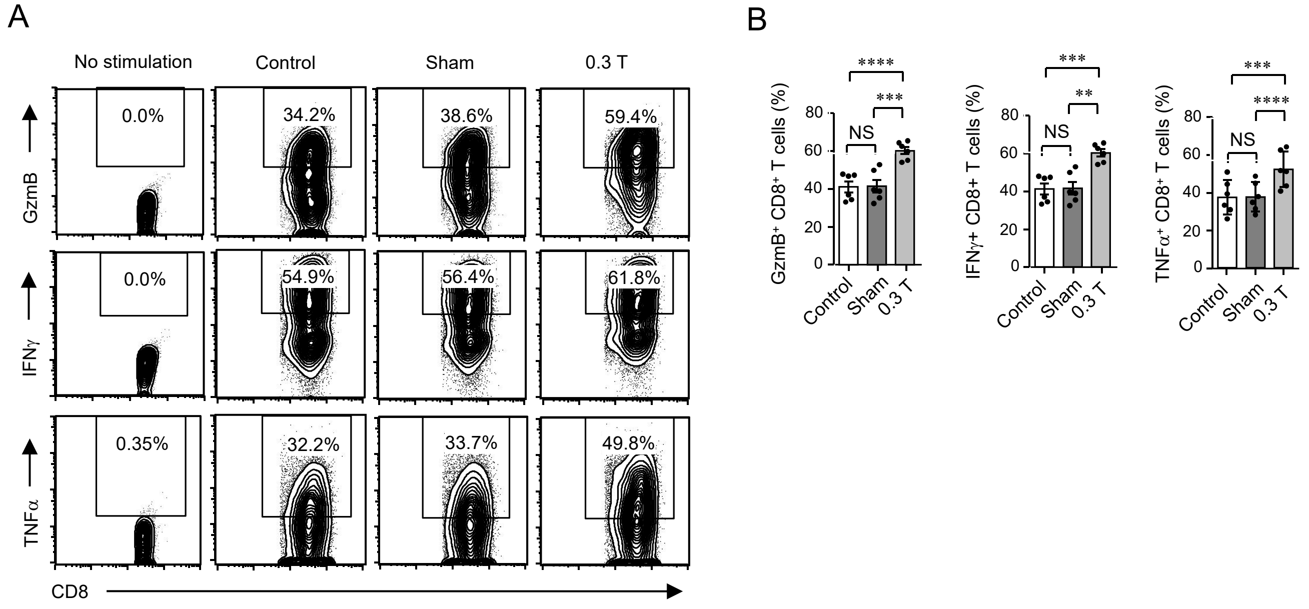

Figure S6. Sham materials have no obvious effects on CD8+ T cell granule and cytokine secretion. (**A**) Cytokine/granule production of stimulated CD8+ T cells analyzed by flow cytometry. Cell samples were stimulated with anti-CD3 and anti-CD28 antibodies for 72 h in the presence of sham materials and 0.3 T permanent magnets, and control cells were treated without magnets. Cell samples with no stimulation were used to show the baseline of cytokine secretion. (**B**) Percentage statistics for the expression of GzmB, IFNγ and TNFα of CD8+ T cells. Data were analyzed by Student's t-test; NS, no significance, **P < 0.01; ***P < 0.001, ****P < 0.0001. Error bars indicate the s.e.m. Data are representative of or combined from at least three independent experiments.

Supplementary Figure 7.

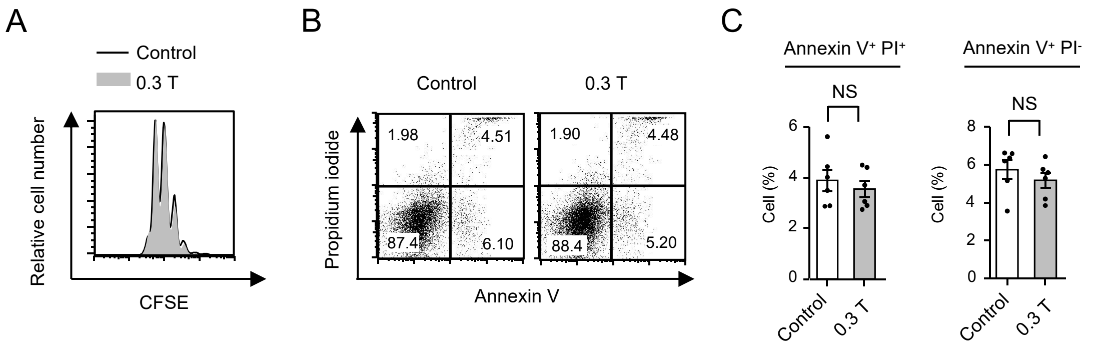

Figure S7. Moderate SMFs do not influence CD8^{+} T cell proliferation and apoptosis.
(**A**) Representative CFSE staining profiles of CD8^{+} T cells stimulated with anti-CD3 and anti-CD28 antibodies for 72h. (**B**) Survival of CD8^{+} T cells stimulated for 72h was assessed by flow cytometry analysis of Annexin V and propidium iodide (PI) staining. (**C**) Percentage statistics for Annexin V and PI staining of CD8^{+} T cells. Cell samples were stimulated in the presence of 0.3 T permanent magnets, and control cells were treated without magnets. Data were analyzed by Student's t-test; NS, no significance. Error bars indicate the s.e.m. Data are representative of or combined from at least three independent experiments.

Supplementary Figure 8.

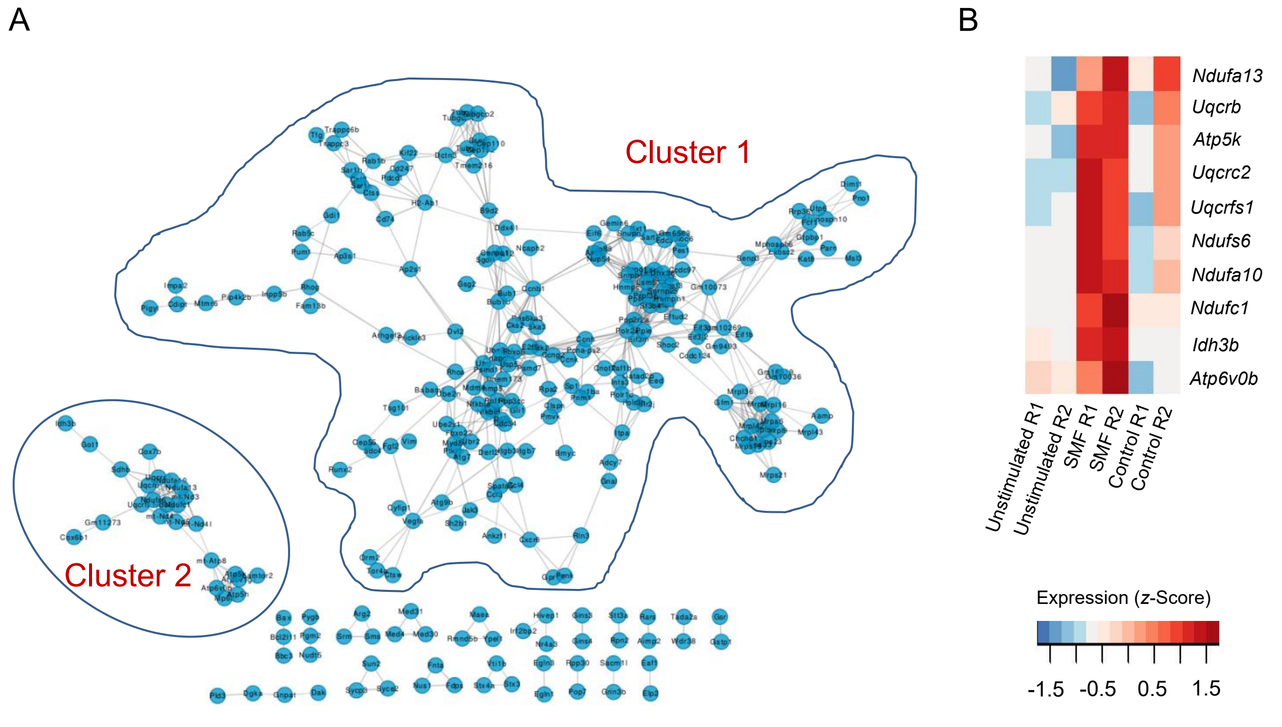

Figure S8. Moderate SMFs promote gene expressions related with mitochondrial respiratory electron transport chain.

(**A**) PPI network (protein protein interaction network) of relative protein expression of differentially expressed genes in 0.3 T SMF-treated CD8$^+$ T cells stimulated with anti-CD3 and anti-CD28 antibodies for 72 h and control cells without magnets. (**B**) A heat map of the relative expression of genes in 0.3 T SMF-treated CD8$^+$ T cells stimulated with anti-CD3 and anti-CD28 antibodies for 72 h (i.e. SMF), control cells stimulated without magnets (i.e. Control), and fresh (unstimulated) cells without magnets.

Supplementary Figure 9.

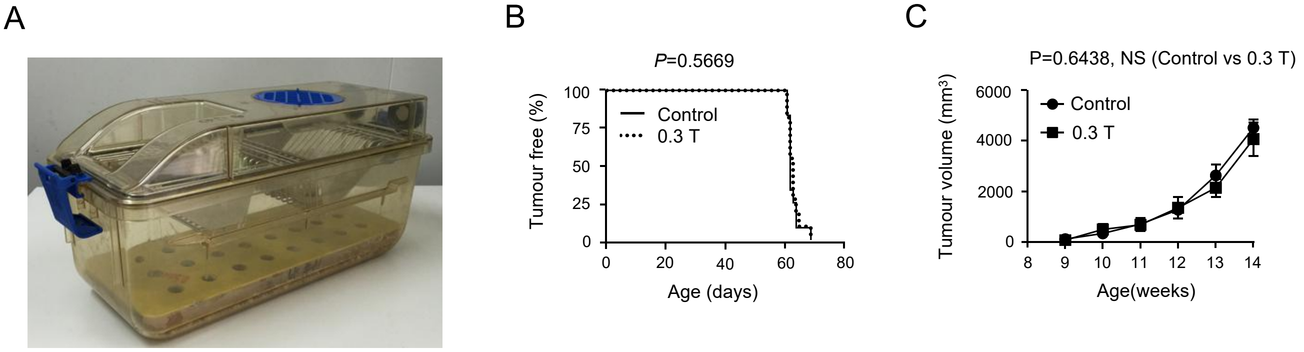

Figure S9. The magnetic plates embedded with 0.3 T magnets have no obvious effects on tumor on set and growth.

(**A**) A magnetic plate was put inside a mouse cage. (**B** and **C**) Tumor onset (**B**; n=11) and tumor growth (**C**; n=7) of PyMT mice exposed to 0.3 T magnetic plates were constantly monitored, and littermates exposed to resin fiber plates without magnets were used as controls. Data were analyzed by log-rank test (**B**) or two-way ANOVA (**C**). NS, no significance. Error bars indicate the s.e.m. Data are representative of or combined from at least three independent experiments.

Supplementary Figure 10.

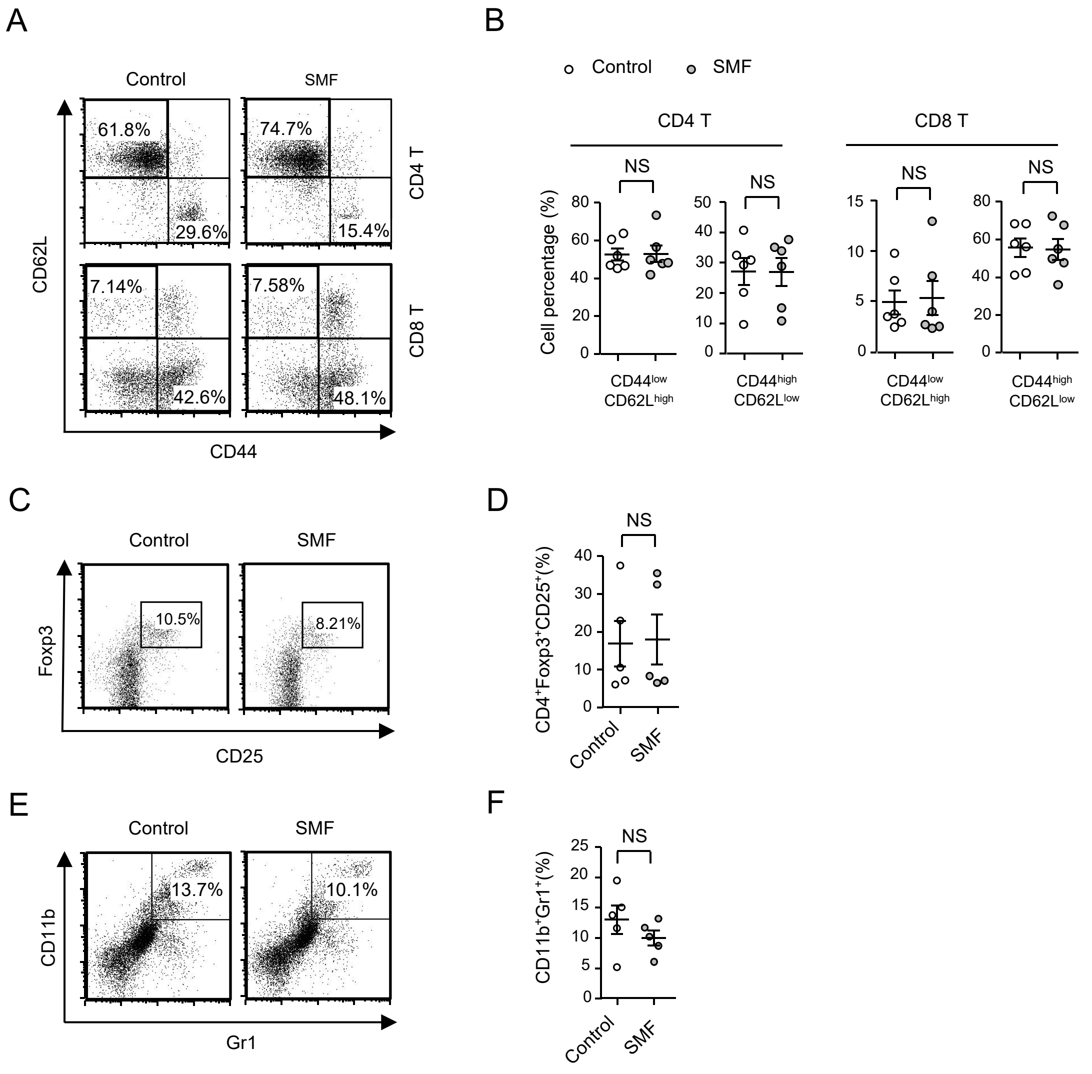

Figure S10. Moderate SMFs do not affect subpopulations of tumour-infiltrating CD8^{+} T cells. (**A**) Expression of CD62L and CD44 of tumour-infiltrating T cells of PyMT mice analyzed by flow cytometry. (**B**) Percentage statistics for CD44^{low} CD62L^{high} and CD44^{high} CD62L^{low} T cells (n=6). (**C**) Flow cytometry analysis of Treg (CD4^{+}CD25^{+} Foxp3^{+}) cells of tumour-infiltrating CD4^{+} T cells from PyMT mice. (**D**) Percentage statistics for Treg cells (n=5). (**E**) Myeloid-derived suppressor cells (MDSC) (Gr1^{+} CD11b^{+}) in the tumour microenvironment were assessed using flow cytometry. (**F**) Percentage statistics for MDSC (n=5). Data were analyzed by Student's t-test; NS, no significance. Error bars indicate the s.e.m. Data are representative of or combined from at least three independent experiments.

Supplementary Figure 11.

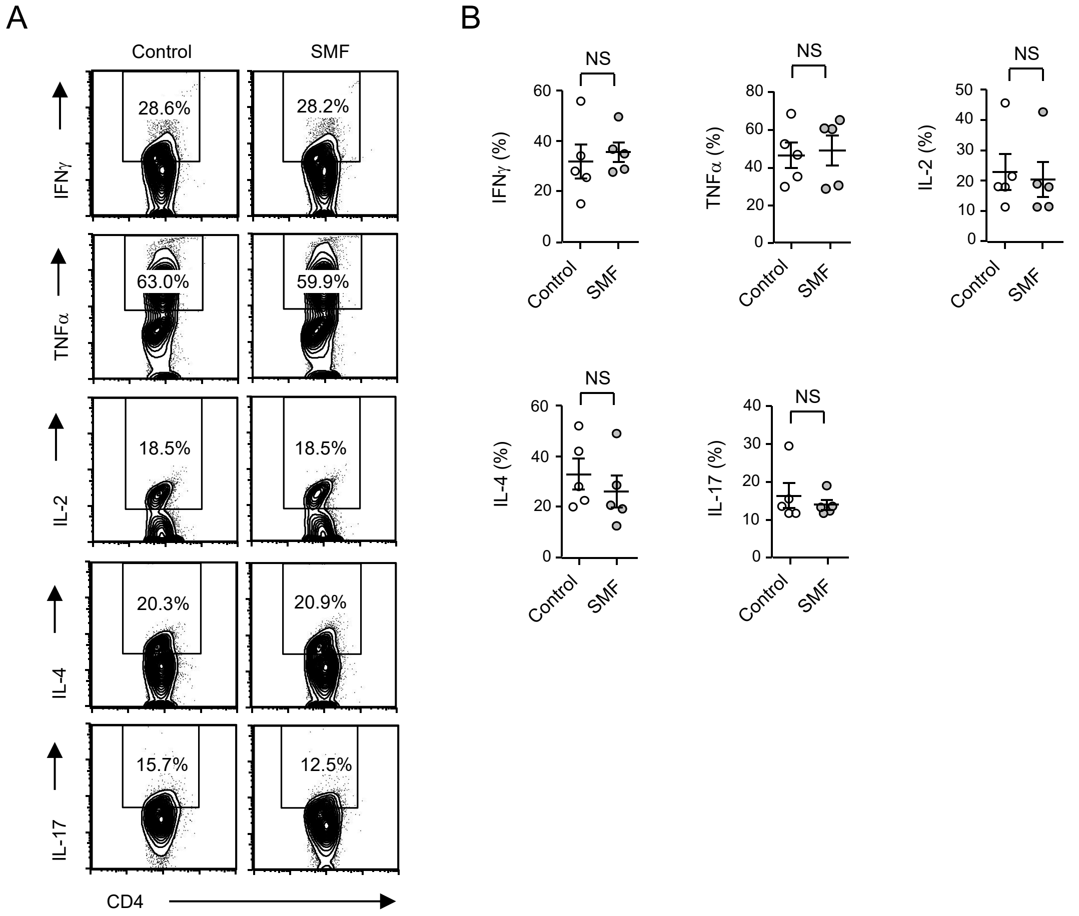

Figure S11. **Moderate SMFs do not influence cell cytokine secretion of tumour-infiltrating CD4$^+$ T cells.**

(**A**) The cytokine productions of tumour-infiltrating CD4$^+$ T cells of PyMT mice were assessed using flow cytometry. (**B**) Percentage statistics for the cytokine productions of tumour-infiltrating CD4$^+$ T cells (n = 5). Data were analyzed by Student's t-test; NS, no significance. Error bars indicate the s.e.m. Data are representative of or combined from at least three independent experiments.
